# Ferroptosis in Liver Disease: Natural Active Compounds and Therapeutic Implications

**DOI:** 10.3390/antiox13030352

**Published:** 2024-03-15

**Authors:** Zhili Wu, Yanru Zhu, Wenchao Liu, Balamuralikrishnan Balasubramanian, Xiao Xu, Junhu Yao, Xinjian Lei

**Affiliations:** 1 College of Animal Science and Technology, Northwest A&F University, Yangling 712100, China; wzl1295354914@163.com (Z.W.); zhuyanru@nwafu.edu.cn (Y.Z.); yaojunhu2004@sohu.com (J.Y.); 2 Key Laboratory of Livestock Biology, Northwest A&F University, Yangling 712100, China; 3 College of Coastal Agricultural Sciences, Guangzhou Ocean University, Zhanjiang 524088, China; liuwc@gdou.edu.cn; 4 Department of Food Science and Biotechnology, College of Life Science, Sejong University, Seoul 05006, Republic of Korea; bala.m.k@sejong.ac.kr; 5 School of Animal Science and Nutritional Engineering, Wuhan Polytechnic University, Wuhan 430023, China; xuxiao200315@163.com

**Keywords:** ferroptosis, natural active compounds, liver disease, therapeutic implications

## Abstract

Ferroptosis is an emerging type of regulated cell death usually accompanied by the accumulation of ferrous ions (Fe^2+^) and lipid peroxides. As the metabolic hub of the body, the liver is crucial for iron storage and lipid metabolism. The liver seems to be closely related to ferroptosis through iron and lipid metabolism. Liver disease greatly threatens host health, and exploring effective interventions is essential. Mounting studies have demonstrated that ferroptosis is one of the possible pathogenic mechanisms involved in liver disease. Targeting ferroptosis may provide a promising opportunity for treating liver disease. However, drugs targeting ferroptosis are extremely limited. Therefore, it is an urgent need to develop new and safe ferroptosis regulators. Natural active compounds (NAC), especially those derived from traditional Chinese medicine, have recently shown great therapeutic potential in liver disease via modulating ferroptosis-related genes or pathways. Here, we outline the molecular mechanism of ferroptosis and systematically summarize the regulatory function of NAC on ferroptosis in liver disease. Finally, we discuss the application prospects and potential problems concerning NAC as ferroptosis regulators for managing liver disease.

## 1. Introduction

Accidental cell death (ACD) and regulated cell death (RCD) are two types of cell death [1]. In general, ACD is generally characterized by virtual immediacy and unpredictability, making it a challenging phenomenon to control [1,2]. However, as a universal and molecularly regulated process in multicellular organisms, RCD is crucial for organism development and homeostasis maintenance, providing a possible way for human intervention in life activities [2,3].

**Figure 1:**
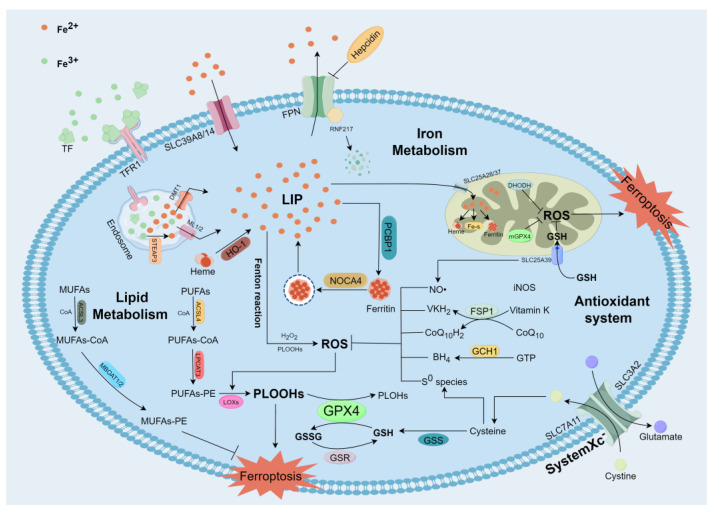
Molecular mechanisms of ferroptosis (by Figdraw). Fe^2+^ accumulation and PUFAs-PE formation are key steps in triggering ferroptosis. Furthermore, some antioxidant systems, including SLC7A11-GSH-GPX4 axis, Cysteine/S^0^ species axis, FSP1-CoQ_10_H_2_/VKH_2_ axis, iNOS/ NO• axis, and GCH1-BH_4_ axis, play a crucial role in preventing phospholipid peroxidation and scavenging reactive oxygen species.

Ferroptosis was first formally proposed in 2012 as an iron-dependent nonapoptotic cell death triggered by erastin, which is a small anticancer molecule targeting RAS mutation-induced cancer cells [4]. The mechanism of ferroptosis has been further expanded in the last decade. A huge molecular regulatory network covering the antioxidant system, iron metabolism, and lipid metabolism has been preliminarily formed [5] (Figure 1).

Liver disease is common and extremely harmful, which greatly burdens the health care system. Approximately 2 million people worldwide die from liver disease every year, accounting for 4 percent of all deaths [6]. Investigating new targets and strategies to treat liver disease is crucial. At the same time, increasing evidence shows that ferroptosis plays a significant role in the progression of liver disease, which provides potential pharmacological targets for liver disease [7]. The therapeutic effects of ferroptosis on liver disease are two-sided. Inhibiting ferroptosis can alleviate liver injury and fatty liver disease. Conversely, promoting ferroptosis can kill hepatic stellate cells (HSCs) and liver cancer cells. In addition, ferroptosis can also influence disease development through nonparenchymal cells in the liver. Exposure to ethanol [8] or *Plasmodium chabaudi* hemozoin [9] could enhance iron uptake by Kupffer cells, which may lead to iron deposition in the liver and induce ferroptosis, accelerating the development of related diseases. Inhibition of ferroptosis of liver sinusoidal endothelial cells during liver cold preservation has also been demonstrated to alleviate injury in orthotopic liver transplantation [10]. Although some anticancer drugs, including cisplatin and sorafenib (SOR), have been shown to induce ferroptosis, they are associated with a range of side effects, such as endocrine dyscrasia, peripheral nerve injury, and bowel dysfunction [11]. Therefore, it is of great interest to develop safe and effective treatment regimens to reduce adverse reactions and drug resistance while treating liver disease via regulating ferroptosis. Given the successful application of artemisinin in treating malaria, natural active compounds (NAC) may open up a broad field for ferroptosis regulation and liver disease treatment [12].

As expected, more and more NAC are useful for direct or adjuvant liver disease treatment through ferroptosis regulation. Many NAC based on traditional Chinese medicine (TCM), such as baicalein, quercetin, and curcumin, are widely present in nature, even in our food, and are characterized by multi-target, structural stability, easy availability, and minimal negative effects [13,14]. Given the lack of a comprehensive overview of advances in using NAC to treat liver disease by targeting ferroptosis, we provide a systemic summary of the therapeutic actions and relevant molecular mechanisms of NAC in the improvement of major ferroptosis-related liver disease and further explore the clinical significance and potential issues.

## 2. The Mechanisms of Ferroptosis

Since ferroptosis was officially proposed in 2012, the search for its mechanism has never stopped. The ferrous ions (Fe^2+^) accumulation triggers the Fenton reaction, producing excess reactive oxygen species (ROS). When the antioxidant system is insufficient to remove these ROS, phospholipid (PL) peroxidation-mediated membrane damage generates many toxic metabolites, eventually leading to ferroptosis [3]. Unlike apoptosis, cells undergoing ferroptosis are usually accompanied by shrunken mitochondrial, increased mitochondrial membrane density and reduction of mitochondrial ridges without the cytoskeleton’s disintegration, nucleoli’s disappearance, and chromosome condensation [4,15]. Meanwhile, the main biochemical features of ferroptosis include the intracellular iron overload, ROS accumulation, and the depletion of endogenous antioxidants such as GSH, which are different from other RCDs. With the rapid development of techniques and methods, a series of key regulatory genes and pathways related to ferroptosis have been identified. In general, the three basic characteristics of ferroptosis are the accumulation of Fe^2+^, PL peroxidation, and imbalance of antioxidant system [5]. Therefore, we summarize the regulatory network of ferroptosis around three factors: iron metabolism, lipid metabolism, and antioxidant system.

### 2.1. Iron Metabolism and Ferroptosis

**Figure 2:**
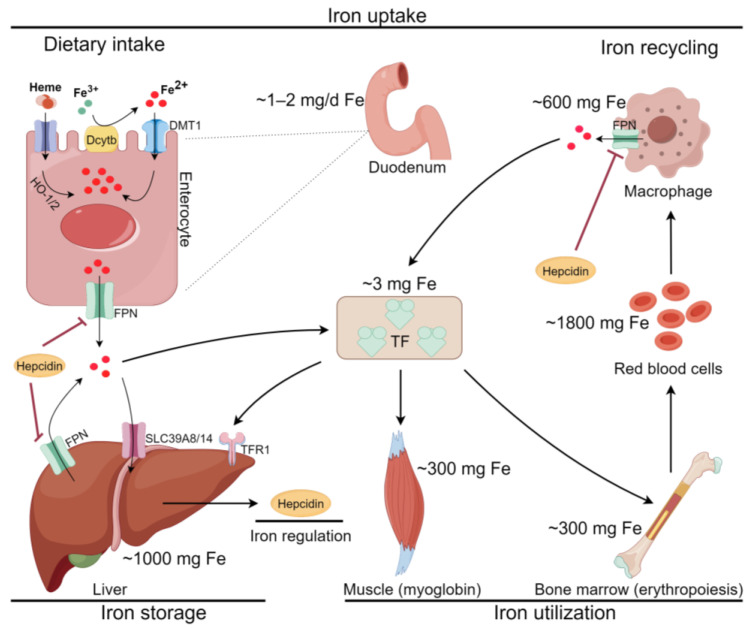
Iron homeostasis in the body (by Figdraw). Fe^3+^ from diet is reduced by Dcytb and then transported into duodenum enterocyte via DMT1. Dietary heme iron is absorbed through an unclear mechanism and decomposed by HO-1/2 in enterocyte. Macrophage degraded red blood cells to recycle iron. The exported iron binds to TF and travels to tissues for utilization. Excess iron can be stored in liver through TFR1 and SLC39A8/14. The release of iron is precisely controlled by FPN, the sole iron exporter. Hepcidin, synthesized by the liver, is the significant regulator of iron homeostasis.

Iron is an indispensable metal element for cell division, metabolism, and growth. It participates in vital physiological activities such as ATP generation, DNA synthesis, and oxygen transport through iron-containing enzymes [16,17,18]. However, excess free iron induces ROS over-production, which is one of the key mechanisms of ferroptosis. Interestingly, even dietary iron content can affect ferroptosis in the liver [19]. Therefore, iron homeostasis is crucial for liver health (Figure 2).

Iron is mainly present as Fe^2+^ or ferric ions (Fe^3+^) in organisms [20]. Duodenal cells that take up iron from the diet and reticuloendothelial macrophages that recover iron from aged erythrocytes are the main suppliers of blood iron, with others provided by body stores such as liver cells, cardiac myocytes, and erythroid cells [18,21]. Approximately one-third of iron is stored in the liver, which makes the liver potentially more sensitive to ferroptosis [22]. After aged erythrocytes are phagocytized by reticuloendothelial macrophages, heme is degraded by heme oxygenase-1 (HO-1), and inorganic iron is recovered [23]. The main form of dietary iron is Fe^3+^, which needs to be reduced to Fe^2+^ by reductases like duodenal cytochrome b (Dcytb) or other non-enzymatic reducing agents before it can be absorbed into the duodenum by divalent metal transporter 1 (DMT1) [16,24]. However, dietary heme iron is absorbed through an unclear mechanism and decomposed into inorganic iron by HO-1 and its homolog HO-2 [23,25,26]. Iron in duodenal cells and reticuloendothelial macrophages enter the blood through ferroportin (FPN); otherwise, it remains in the cytoplasm in certain forms such as ferritin, which is made up of ferritin light chain (FTL) and ferritin heavy chain 1 (FTH1) [17,27]. Hepcidin, a hormone synthesized and secreted primarily by liver cells, binds to FPN and induces its internalization and degradation via E3 ubiquitin-protein ligase RNF217, thereby preventing excessive iron levels in the circulatory system [18,28]. The exported Fe^2+^ is rapidly oxidized to Fe^3+^ by ceruloplasmin or hephaestin [23]. Two Fe^3+^ ions are bound to transferrin (TF) in plasma and enter iron-requiring cells via transferrin receptor 1 (TFR1)-mediated endocytosis [23,29]. In a recent study, TF nanovesicles coupled with Fe^3+^ and encapsulated SOR (SOR@TF-Fe^3+^ NVs) were developed to accelerate iron transport and enhance SOR efficacy. SOR@TF-Fe^3+^ NVs could more efficiently promote the production of lipid peroxides (LPO), suppress tumor growth, and prolong survival rates in hepatocellular carcinoma (HCC) mouse model than SOR or TF- Fe^3+^ NVs alone [30]. In endosomes, Fe^3+^ is released from TF and then reduced to Fe^2+^ via the six-transmembrane epithelial antigen of prostate 3 (STEAP3) [31]. Later, Fe^2+^ enters the labile iron pool (LIP) through DMT1 or mucolipin 1/2 (ML1/2) [7,32]. In addition, HO-1-mediated heme degradation, solute carrier family 39 member 8/14 (SLC39A8/14)-mediated Fe^2+^ import, and nuclear receptor coactivator 4 (NCOA4)-mediated ferritinophagy can increase LIP amount [33]. However, the role of HO-1 in ferroptosis requires further research. In AML12 and HepG2 cells, HO-1 knockdown could accelerate ROS accumulation, lipid peroxidation, and iron overload. In contrast to HO-1 overexpression, HO-1 knockdown also decreased glutathione (GSH) and superoxide dismutase (SOD) levels in vitro [34]. This result may be associated with the HO-1 mediated-enhancement of glutathione peroxidase 4 (GPX4) activity [35]. There are four main destinations of Fe^2+^ in LIP [5,7]: (1) Fe^2+^ is exported to extracellular space through FPN; (2) Fe^2+^ is used in the synthesis of iron-containing proteins; (3) Fe^2+^ binds to ferritin through the iron chaperone poly (rC) -binding protein 1 (PCBP1); and (4) Fe^2+^ enters mitochondria via SLC25A28/37 for heme, mitochondrial ferritin, and iron-sulfur (Fe-S) clusters synthesis. Two pathways mainly regulate the intracellular iron metabolism [32]. One is the iron acquisition pathway mediated by the iron regulatory protein1/2 (IRP1/2) [36,37], and the other is the tristetraprolin-mediated iron conservation pathway [38]. Similarly, both pathways regulate intracellular iron levels through the interaction of certain transfer factors with the mRNA of iron metabolism-related proteins such as TFR1, FPN, and FTH1. It is important to note that the regulatory mechanisms of iron metabolism are quite complex, and the above described are only the most basic aspects. The dysfunction of any process of cellular iron metabolism may lead to Fe^2+^ overload in LIP. Unstable Fe^2+^ triggers the Fenton reaction to generate excess ROS, leading to PL peroxidation. The substrate of the Fenton reaction can be either hydrogen peroxide (H_2_O_2_) or phospholipid peroxides (PLOOHs) [39]. Fe^2+^ can also promote PL peroxidation by increasing the activity of lipoxygenases (LOXs), nicotinamide adenine dinucleotide phosphate (NADPH) oxidases (NOXs), and EGLN prolyl hydroxylases [3,29,40]. When PLOOHs cannot be removed promptly and effectively, the integrity of the cell membrane will be destroyed, and ferroptosis will eventually occur.

### 2.2. Lipid Metabolism and Ferroptosis

PL peroxidation is one of the key factors driving ferroptosis. Although PL peroxidation is only one part of lipid metabolism, increasing research suggests that lipid metabolism can influence ferroptosis through multiple dimensions. As a hub of lipid metabolism, the liver is essential for lipid synthesis, storage, consumption, and transportation. The liver may produce more ROS during lipid metabolism, increasing ferroptosis sensitivity. Fatty acids (FAs) can be obtained directly from the diet or synthesized de novo in cells. Free FAs can enter cells via passive diffusion or membrane-associated proteins such as clusters of differentiation 36. Meanwhile, FAs contained in some lipoproteins can be endocytosed via related receptors, such as very low-density lipoprotein receptors [41]. Interestingly, the type of FAs in the diet appears to change the composition of FAs in cells, thereby altering the sensitivity of cells to ferroptosis. When rat β-cells were treated with long-chain saturated fatty acids (SFAs) and ω-6 polyunsaturated fatty acids (PUFAs), both resulted in PL peroxidation, but only the latter triggered ferroptosis [42]. In an acidotic environment, ω-3 and ω-6 PUFAs selectively induced ferroptosis in cancer cells. Notably, a diet rich in long-chain ω-3 PUFAs significantly delayed tumor growth in mice compared with monounsaturated fatty acids (MUFAs)-rich diet [43]. This provides implications for dietary therapies targeting ferroptosis. As essential fatty acids, long-chain ω-3 and ω-6 PUFAs can only be obtained from diet, but both SFAs and MUFAs can be synthesized de novo in cells [39]. Generally, SFAs and MUFAs are less sensitive to ferroptosis than PUFAs. Inhibition of key enzymes for the synthesis of MUFAs, such as stearoyl-CoA desaturase 1 (SCD1), could significantly enhance the anti-tumor effects of ferroptosis inducers in ovarian cancer cell lines and mouse orthotopically xenograft models [44]. While knockout of fatty acid desaturases (FADSs) involved in PUFAs metabolism, such as FADS2, could protect immortalized primary hepatocytes (PH5CH8) and lung cancer cells (A549) from ferroptosis induced by erastin [45]. β-oxidation is thought to inhibit ferroptosis by depleting PUFAs. Fatty acid binding protein 4 (FABP4) plays a vital role in β-oxidation as it transports free FAs to mitochondria and peroxisomes. Under high glucose conditions, inhibition of FABP4 could make human renal proximal tubular epithelial (HK2) cells more sensitive to ferroptosis by inhibiting β-oxidation, which may provide a new therapeutic strategy for treating diabetic kidney disease [46]. FAs must be conjugated to coenzyme A (CoA) by the long-chain acyl-CoA synthetases (ACSLs) family before they can be esterified into PLs. Interestingly, ACSL3 preferred MUFAs as the substrate, while ACSL4 preferred PUFAs such as arachidonic acid (AA) and adrenic acid (AdA) [47]. This implies that regulating the activity of ACSLs may affect the membrane components and thus alter the sensitivity of cells to ferroptosis [48]. Ferroptosis is crucial to irradiation (IR)-induced intestinal injury, and ACSL4 is highly expressed in irradiated intestinal tissues. A recent study showed that ACSL4 inhibitor (troglitazone) could inhibit intestinal PL peroxidation and tissue damage after IR [49]. However, when ACSL4 was hepatocyte-specific deleted in mice, there was no increase in HCC, and the liver showed less fibrosis and proliferation, especially in the HCC model of toxic injury induced by diethylnitrosamine and carbon tetrachloride (CCl_4_) [50]. This implies that the anticancer effect of ferroptosis in HCC is not absolute. FAs-CoA are then incorporated into membrane PLs by the lyso-phosphatidylcholine acyltransferases (LPCATs) family or lyso-phosphatidyl CoA acyltransferases [39]. In this process, LPCAT3 prefers to esterify AA/AdA-CoA to PUFA-phosphatidylethanolamines (PEs), the key PLs that trigger ferroptosis [11]. Because LPCAT3 inhibitors can change the content of PUFA-PLs in cells and protect from ferroptosis, LPCAT3 is considered as a determinant of ferroptosis [51]. Membrane-bound O-acyltransferase domain 1/2 (MBOAT1/2) are phospholipid-modifying enzymes that the androgen receptor and estrogen receptor can directly upregulate. Interestingly, it has been demonstrated that MBOAT1/2 could selectively transfer MUFAs to lyso-PEs, thereby reducing the sensitivity to ferroptosis [52]. Both LOXs-based enzymatic reaction and free Fe^2+^-based non-enzymatic reaction promote PL peroxidation. In CCl_4_-induced acute liver injury (ALI), arachidonate 15-lipoxygenase (ALOX15) triggered PL peroxidation, but genipin treatment could attenuate this process [53]. It should be emphasized that the enzymatic activity of LOXs may not be universally required for ferroptosis. Cyclooxygenases, cytochrome P450, and NOXs can also promote PL peroxidation [54]. PL peroxidation is a process that can propagate and in turn produce more PLOOHs until the reaction is terminated. Ferroptosis may occur when the antioxidant system represented by GPX4 is insufficient to remove ROS, especially PLOOHs.

### 2.3. Antioxidant System and Ferroptosis

If Fe^2+^ overload and PL peroxidation are the arsonists of ferroptosis, the antioxidant system is undoubtedly the firefighter of this fire. Various antioxidant protective mechanisms can timely remove ROS or terminate the transmission of PL peroxidation, thereby protecting cells against ferroptosis. GPX4 is identified as a key negative regulator of ferroptosis by reducing toxic PLOOHs to non-toxic phospholipid alcohols (PLOHs) [55,56]. The inactivation of GPX4 is sufficient to cause uncontrolled membrane lipid peroxidation, leading to ferroptosis in multiple organs and abnormal body development [57]. GSH, a tripeptide composed of glutamate, cysteine and glycine, is mainly synthesized in liver. As an essential substrate for GPX4, GSH is oxidized to oxidized glutathione (GSSG). Then, GSSG can be reduced to GSH through glutathione-disulfide reductase (GSR) with NADPH as the electron donor [54]. Cystine-glutamate antiporter (System Xc^−^), consisting of SLC7A11 and SLC3A2, is an important pathway for cells to obtain cysteine, a key substrate for GSH synthesis. Inactivation of GPX4 by disrupting System Xc^−^ has been demonstrated to treat various diseases. For example, the anticancer molecule SOR could promote ferroptosis of HSCs by reducing SLC7A11 to alleviate liver fibrosis (LF) [58]. In addition, nuclear factor erythroid 2-related factor 2 (NRF2) and heat shock protein family A member 5 can act as positive regulators of GPX4. At the same time, P53 and many non-coding RNAs, such as miR-539 and miR-6516-5p, can down-regulate GPX4 [59]. They have all become important targets for the regulation of ferroptosis. Paralleling the GPX4-based mechanism, endogenous lipophilic radical-trapping antioxidants (RTAs) such as ubiquinol (CoQ_10_H_2_), vitamin K hydroquinone (VKH_2_), and dihydrobiopterin (BH_4_) can also prevent ferroptosis by scavenging lipid free radicals [60]. Ferroptosis suppressor protein 1 (FSP1) is a NAD(P)H-ubiquinone reductase that is located in the plasma membrane. It can reduce ubiquinone (CoQ_10_) to CoQ_10_H_2_, which in turn prevents the proliferation of LPO [61,62]. FSP1 can effectively reduce vitamin K to VKH_2_, including menadione and phylloquinone, exerting its anti-ferroptosis function [63]. The recently identified FSP1 inhibitor 3-phenyl quinazolinones, represented by icFSP1, could induce FSP1 agglutination in tumors and synergize with ferroptosis inducers to enhance the ferroptosis response, thereby inhibiting tumor growth in vivo [64]. GTP cyclohydrolase 1 (GCH1) is the rate-limiting enzyme for the synthesis of BH_4_ [65]. Interestingly, BH_4_ not only can act as a direct antioxidant to prevent cells from lipid peroxidation, but also can be used to synthesize CoQ_10_ de novo, which is equivalent to providing a double safeguard against ferroptosis [66]. As the main organelle for ROS production in cells, the mitochondrion is closely associated with ferroptosis, and their relationship has attracted much attention. As expected, mitochondria have their mitochondrial GPX4 (mGPX4)-independent antioxidant pathways. Dihydroorotate dehydrogenase (DHODH) is a flavin-dependent enzyme in the inner mitochondrial membrane. A recent study found that DHODH can not only oxidize dihydroorotate to orotate, but also reduce COQ_10_ to COQ_10_H_2_ [67]. Together with mGPX4, DHODH constructs the antioxidant system of mitochondria, which withstands the tremendous pressure of mitochondrial membrane lipid peroxidation. Uridine, a key substrate for synthesizing DNA, RNA, and glucose, was shown to trigger ferroptosis in HCC cells and suppress the further development of HCC [68]. Uridine synthesis in tumor cells mainly depends on the de novo synthesis pathway [69]. Interestingly, DHODH is one of the rate-limiting enzymes for de novo synthesis of uridine and its expression can be inhibited by high concentration of uridine in vitro, which may explain why uridine can induce ferroptosis in HCC cells [68]. However, the inhibitory effect of DHODH on ferroptosis is controversial because DHODH only functions at high concentrations that also effectively inhibit FSP1 [70]. Sulfane sulfur (S^0^) species have a potent antioxidant effect. Still, their relationship with ferroptosis was unclear in the past [71,72]. In addition to synthesizing GSH, cysteine can also be used to synthesize S^0^ species, but the intracellular concentration of S^0^ species is much lower than that of GSH. However, it has been demonstrated that the S^0^ species, especially hydropersulfides, could stop radical chain reactions via the formation and self-recombination of perthiyl radicals, thereby inhibiting lipid peroxidation and ferroptosis [73]. Given its prevalence in living organisms, hydropersulfides may represent a primitive radical scavenging system [74]. Previous studies have shown that depletion of cystathionine β-synthase (CBS), an enzyme promoting hydropersulfide synthesis by providing H_2_S, could sensitize breast cancer cells to ferroptosis without affecting GSH levels [75]. This suggests that modulation of substances that affect hydropersulfides may be an attractive strategy for anti-HCC chemotherapy [74]. Furthermore, exogenous hydropersulfides donors have been shown to inhibit ferroptosis in various cell models, implying that the development of hydropersulfides donors with optimal drug-like properties and selectivity for specific tissues could be a potential therapeutic strategy for liver disease [76]. In addition, the nitroxygenation of inducible nitric oxide synthase (iNOS)-derived NO• with 15-LOX-generated lipid intermediates such as eicosatetraenoyl-PE could enhance resistance to ferroptosis [77].

It is worth mentioning that the whole-genome CRISPR activation screen has played a significant role in discovering key antioxidative enzymes and pathways. We believe that with the development of related technologies, more anti-ferroptosis mechanisms will be discovered, bringing more opportunities for treating ferroptosis-related diseases.

## 3. NAC Treat Liver Disease by Targeting Ferroptosis

**Figure 3:**
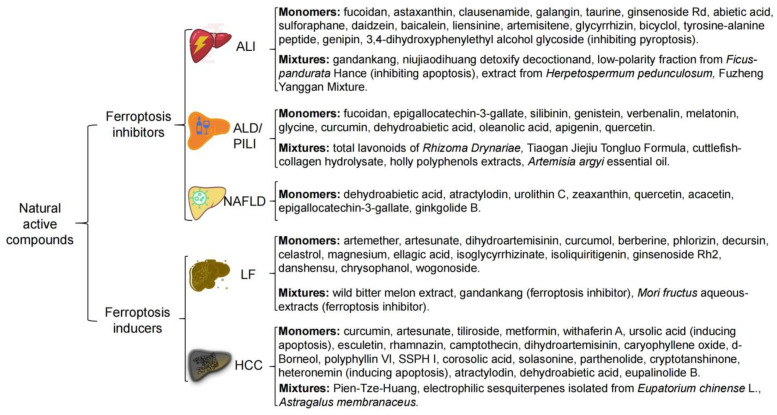
Natural active compounds exert therapeutic effects on liver disease by regulating ferroptosis. ALI, acute liver injury; ALD, alcohol-related liver disease; PILI, pollutants-induced liver injury; NAFLD, non-alcoholic fatty liver disease; LF, liver fibrosis; HCC, hepatocellular carcinoma.

Liver disease is one of the major threats to human health. Increasing evidence shows that ferroptosis is closely related to liver disease [7,78,79,80]. Currently, there are no definitive effective drugs for the improvement of liver disease, such as ALI, non-alcoholic fatty liver disease (NAFLD), and LF. In addition, drug resistance in cancer treatment has always been an issue at present [13]. Therefore, it is urgent to develop safe and effective drugs. NAC, including TCM, have become an important source for the development of drugs targeting ferroptosis due to their high therapeutic potential and low toxicity. Among them, various flavonoids and terpenoids have been reported to exert certain curative effects on liver disease via targeting ferroptosis [13,14]. Here, we summarize the therapeutic impact of newly discovered NAC on treating main liver disease and the potential mechanisms of regulating ferroptosis (Figure 3).

### 3.1. Acute Liver Injury (ALI)

ALI is a clinical syndrome of liver failure caused by rapid damage of hepatocytes in the absence of pre-existing cirrhosis [81]. ALI, as a rare and life-threatening disease, can be caused by drugs, viruses, ischemia, or other external reasons [82,83]. Acetaminophen (APAP) is widely used for relieving heat and pain, but its overuse can cause ALI. Previous studies have demonstrated that ferroptosis is related to APAP-induced liver injury [84,85]. *Nrf2* is a key gene against ferroptosis by regulating a series of proteins related to iron metabolism, antioxidants, and autophagy, such as FTH1, GPX4, SLC7A11, and HO-1 [83,86]. Interestingly, fucoidan [87], abietic acids [88], astaxanthin [89], clausenamide [90], daidzein [91], 3,4-dihydroxyphenylethyl alcohol glycoside [92], Fuzheng Yanggan Mixture [93], and water extract from *Herpetospermum pedunculosum* [94] could alleviate APAP-induced liver injury by regulating *Nrf2* or downstream effector proteins. In liver transplantation, ischemia-reperfusion (I/R) usually causes ALI, and ferroptosis is the therapeutic target to alleviate I/R injury [95]. The phosphatidylinositol-3-kinase (PI3K)/protein kinase B (AKT) pathway was crucial to regulating cell death [96]. Meanwhile, cAMP response element-binding protein (CREB) has been reported to promote GPX4 expression [97]. Galangin, a natural flavonoid, has been shown to exert its anti-ferroptosis effect, possibly by activating the PI3K/AKT/CREB pathway, significantly improving the pathological damage of liver tissues in mice with I/R [98]. Taurine, widely present in marine animal tissues, is a potential drug for alleviating I/R injury in liver due to its excellent antioxidant and anti-inflammatory properties. Recently, taurine has been shown to increase the expression of GPX4 and SLC7A11 in liver tissues, which suggests the anti-ferroptosis effect of taurine and its therapeutic potential in liver I/R injury [99]. CCl_4_ and lipopolysaccharide (LPS)/D-galactosamine (D-gal) are often used as inducers in the construction of the ALI model. TCM and its derivatives have shown great therapeutic potential for ALI. Although the mechanisms are not exactly the same, ginsenoside Rd [100], bicyclol [101], gandankang [102], sulforaphane [83], genipin [53], baicalein [103], liensinine [104], artemisitene [105], glycyrrhizin [106], niujiaodihuang detoxify decoction [107], and low-polarity fraction from *Ficus pandurata* Hance [108] have all been shown to alleviate CCl-4 or LPS/ D-gal-induced ALI via preventing ferroptosis. NRF2, GPX4, and lipid metabolism-related enzymes such as ALOX12/15 and ACSL4 are the major targets of these TCMs. The cyclic GMP-AMP synthase (cGAS)/stimulator of interferon genes (STING) pathway is crucial for the immune system [109]. Recent studies have shown that the cellular redox homeostasis maintained by GPX4 is required for STING activation [77], and that cGAS inhibits ROS excessive accumulation by promoting the oligomerization of dynamin-related protein 1 in the outer mitochondrial membrane [110]. Interestingly, ginsenoside Rd alleviated CCl_4_-induced ALI in mice by inhibiting ferroptosis through the cGAS/STING pathway, which further implies the therapeutic potential of TCM for ALI by targeting ferroptosis [100]. Bioactive peptides can be used as therapeutic agents for many diseases due to their diverse biological functions. Tyrosine-alanine (YA) peptide, the main ingredient of oyster-derived hydrolysate, possesses strong antioxidant and anti-inflammatory properties. A recent study showed that YA pretreatment could reverse the ferroptosis in LPS/D-gal-induced ALI model and also prevent ALI by inhibiting inflammatory, apoptosis, and pyroptosis [111].

### 3.2. Alcohol and Environmental Pollutants-Induced Liver Disease

In daily life, alcohol consumption is an important cause of liver injury. Alcohol-related liver disease (ALD), the leading global cause of chronic liver disease, involves pathological processes ranging from hepatic steatosis to inflammation, fibrosis, cirrhosis, and HCC [112]. Increasing evidence suggests that ferroptosis plays an important role in ALD and holds promise as an ideal target [113]. Alcohol promotes intestinal iron absorption and increases the risk of hepatic iron overload through a synergistic effect with free iron [113]. In addition, acetaldehyde, the major intermediate metabolite of ethanol, is responsible for the generation of ROS and down-regulating the expression of key antioxidant genes such as *Nrf2*, thereby impairing the antioxidant system [114]. As the most potent active component of tea polyphenols, epigallocatechin-3-gallate (EGCG) may prevent and treat ALD. On the one hand, EGCG could alleviate hepatic iron overload by inhibiting intestinal absorption of non-heme iron and upregulating the expression of FTH1 and FTL. On the other hand, EGCG upregulated NRF2 and GPX4 expression and improved antioxidant function in mice suffering from iron overload [115]. Fucoidan, a polysaccharide derived from brown algae, is a natural antioxidant because of its sulfuric acid group [116]. Similar to EGCG, fucoidan could inhibit hepatic iron overload via regulating hepcidin-intestinal DMT1/FPN axis and alleviate oxidative damage of liver cells through upregulating P62/NRF2/SLC7A11 pathway in rats that were exposed to alcohol for a long term [117]. Silibinin and genistein, both natural flavonoids, could alleviate ethanol-or acetaldehyde-induced liver injury via inhibiting NCOA4-mediated ferritinophagy and activating NRF2/HO-1 pathway, respectively [114,118]. PTEN-induced putative kinase 1(PINK1)/Parkin-mediated mitophagy could suppress intracellular ROS accumulation by removing damaged mitochondria [119], but its relationship with ferroptosis is still unclear. A recent study reported that silibinin could bind to PINK1 and Parkin directly, promote PINK1/ Parkin-mediated mitophagy, and reduce ferritin degradation as well as ROS levels, thereby protecting against ferroptosis [118]. Furthermore, silibinin may directly bind to TFR1 to inhibit cellular iron uptake and maintain iron homeostasis in ethanol- and acetaldehyde-induced liver injury [118]. Both murine double minute X (MDMX)/peroxisome proliferator-activated receptor alpha (PPARα) pathway [120] and liver kinase B1 (LKB1)/AMP-activated protein kinaseα (AMPKα) signal axis [121] were shown to be associated with ferroptosis. For alcohol-induced liver injury, verbenalin [120] and Tiaogan Jiejiu Tongluo Formula [121] showed a certain therapeutic effect through inhibiting intracellular lipid peroxidation, which was regulated by the MDMX/PPARα pathway and LKB1/AMPKα signal axis, respectively. Melatonin, a hormone present in various organisms from algae to humans, is responsible for regulating circadian rhythms and is also an important endogenous antioxidant. Brain and muscle ARNT-like 1 (BMAL1) is a circadian clock protein found to promote ferroptosis through autophagic degradation of itself [122]. A recent study showed that melatonin exerted its anti-ferroptosis effect by activating the BMAL1-dependent activation of NRF2-related antioxidant response elements (ARE) [123]. At present, environmental pollutants such as heavy metals, non-metallic toxic elements, and pesticides are also important inducements of liver injury. Therefore, it is important to explore the NAC against the pollutants-induced liver injury (PILI). Diquat is a selective herbicide that can induce oxidative stress, karyolysis, karyopyknosis, and changes in hepatic cord arrangement in piglets [124]. Recent studies showed that holly polyphenols extracts (HPE) [124] and glycine [125] could alleviate diquat-induced liver injury by targeting ferroptosis. Mechanistically, they both enhanced GPX4 expression, and HPE also inhibited the transfer of Fe^3+^ by decreasing TFR1 abundance [124,125]. Bisphenol A (BPA), an environmental pollutant used in manufacturing plastic packaging materials, was reported to disrupt lipid metabolism and promote ferroptosis in the liver by activating the G protein-coupled estrogen receptor. *Artemisia argyi* essential oil, a volatile oil component extracted from leaves of *Artemisia argyi H. Lév. & Vaniot*, was shown to increase GPX4 expression and reduce the accumulation of Fe^2+^ in cells, thereby alleviating BPA-induced liver ferroptosis [126]. Fluoride is a toxic non-metallic element, and liver is considered one of the important target organs of fluorosis. The silent information regulator 1(SIRT1)/forkhead box O3 (FOXO3) pathway was involved in rats’ aluminum phosphide-induced acute lung injury [127]. Similarly, the SIRT1/FOXOs pathway could lead to lipid peroxidation and iron accumulation under fluorosis conditions, ultimately triggering ferroptosis [128]. Alpha lipoic acid is an important natural free radical scavenger. It was reported to inhibit the occurrence of lipid peroxidation via the System Xc^-^/GPX4 axis, thereby preventing fluorine-induced ferroptosis in liver cells [129]. Alpha lipoic acid also ameliorated cobalt-induced liver injury via inhibiting ferroptosis [130]. In addition, ammonia [131], lead [132], mercuric chloride [133], ethyl carbamate [134,135], di (2-ethylhexyl) phthalate [136], aflatoxin B1 [137], and acrylamide [138] could cause liver injury by inducing ferroptosis. And there are corresponding NAC to prevent or treat pollutants-induced liver injury (Table 1).

### 3.3. Non-Alcoholic Fatty Liver Disease (NAFLD)

The NAFLD, first proposed by Schaffner in 1986, is characterized by excessive accumulation of liver fat and defined as the presence of steatosis in 5% of hepatocytes histologically [139]. Due to its close association with metabolic diseases, NAFLD has been proposed to be replaced by metabolic fatty liver disease, which can more accurately reflect the pathogenesis of metabolic dysfunction and fatty liver disease in patients [140,141]. We habitually use NAFLD throughout this review. As the most common liver disease in the world, NAFLD has a global prevalence of about 30% [139]. NAFLD is characterized by liver cell injury, liver cell death, inflammation, oxidative stress, insulin resistance, and lipid metabolism disorders [141,142]. Meanwhile, the pathogenesis of NAFLD also involves endoplasmic reticulum (ER) stress, mitochondrial dysfunction, genetic susceptibility, and gut-liver axis related signal transduction [141,143]. The NAFLD covers a spectrum of liver disorders ranging from simple fatty accumulation in the liver to the more severe form of steatohepatitis, which may eventually progress to life-threatening cirrhosis and HCC [142]. However, the mechanism of NAFLD is not fully understood, and there is currently no recognized standard therapy for the treatment of NAFLD. Iron overload is common in patients with NAFLD, and iron-induced lipid peroxidation is an important factor in NAFLD [80]. Malondialdehyde (MDA) and 4-hydroxynonenal (4-HNE) are the products of lipid peroxidation. Interestingly, MDA and 4-HNE were increased in more than 90% of patients with NAFLD [144]. Oxidative stress is considered to be the main factor in the development of steatosis to non-alcoholic steatohepatitis (NASH). The disorder of iron metabolism is also an important feature of NASH [145]. RSL3 (ferroptosis activator) was shown to aggravate NASH symptoms, which were alleviated by sodium selenite (GPX4 activator) and deferoxamine mesylate salt (iron chelator) [146]. As an important ferroptosis regulator, NRF2 was shown to be down-regulated in NAFLD mice, and enhancing the NRF2/HO-1 pathway could effectively prevent the development of NAFLD [147,148]. Accumulating evidence suggests that ferroptosis can induce oxidative stress, aggravate inflammation, and promote cell damage, thereby accelerating the pathological process of NAFLD [22]. Given the close association between ferroptosis and NAFLD, exploring several potential NAC targeting ferroptosis to treat NAFLD is necessary.

Dehydroabietic acid (DAA) is a natural diterpene with anti-tumor [149], anti-inflammatory [150], anti-bacteria [151], and other biological activities. Under normal physiological conditions, kelch-like epichlorohydrin-related protein-1 (KEAP1) binds to NRF2 in the cytoplasm and inactivates NRF2 [152]. A previous study showed that DAA could improve hepatic steatosis induced by high-fat diet (HFD) through activating PPAR-γ and PPAR-α [153]. Further research showed that DAA could release NRF2 after binding to KEAP1 and suppress ferroptosis via regulating the NRF2-ARE pathway, thus improving HFD-induced NAFLD [152]. Atractylodin (ART) is a natural active component extracted from *Atractylodes lancea De Candolle*, with pharmacological properties such as anti-oxidation and anti-inflammation actions [154]. Ginkgolide B (GB), a terpene trilactone extracted from of *Ginkgo biloba* leaves, has anti-spinal cord injury and neuroprotective effects [155]. Similar to DAA, GB [155] and ART [154] could inhibit ferroptosis through the NRF2 pathway, thereby alleviating oxidative stress in NAFLD. Urolithin C (UroC) is one of ellagitannin’s most abundant bioavailable gut microbiota metabolites and contains two phenolic rings with o-dihydroxyl and mono-hydroxyl substitutions [156]. Gut microbiota and the liver may interact through the gut-liver axis. Microbiota disorder is one of the main characteristics of NAFLD. Studies about microbiota transplantation suggest adjusting microbiota disorders may be an effective measure for the treating NAFLD [157]. A recent study demonstrated that UroC could normalize the *Firmicutes* to *Bacteroidota* ratio and increase the ratio of some beneficial bacteria such as *Parabacteroides goldsteinii* and *Lactobacillus vaginalis* in NAFLD mice induced by choline-deficient, amino acid-defined and high-fat diet [156]. Mechanistically, UroC may regulate lipid metabolism by activating the AMPK pathway to inhibit ferroptosis, thereby alleviating NAFLD [156]. Zeaxanthin (ZEA), a carotenoid from the isoprene group, is widely found in green leafy vegetables, fruits, and yellow corn. P53 protein is not only an important tumor suppressor but also a regulator of ferroptosis. P53 could inhibit the expression of SLC7A1, resulting in decreased GSH biosynthesis and GPX4 activity [158]. Meanwhile, P53 could also upregulate ALOX15 and induce lipid peroxidation by activating spermidine/spermine N1-acetyltransferase 1 [159]. ZEA could down-regulate the expression of P53 in free FA-induced HepG2 cells, thereby reducing cellular lipid peroxidation and inhibiting ferroptosis, suggesting that ZEA has the potential to intervene NAFLD [160]. Mitochondrion, as the main site of ROS production, is closely related to ferroptosis, and plays a vital role in the development of NAFLD [161,162]. A previous study confirmed that mitochondrial ROS (mROS) could aggravate hepatocyte oxidative damage and promote NAFLD development [163]. Previous studies have demonstrated that EGCG [164] and quercetin [165] both alleviated lipid accumulation stress in HFD-induced steatotic hepatocytes by targeting mROS-mediated ferroptosis. In addition, EGCG could also improve intestinal microbiota dysbiosis and certain enzymes from genera to affect host metabolism, thereby protecting against NASH induced by methionine-choline-deficient diet. ER is the main site of lipid synthesis in hepatocytes. ER stress may cause lipid metabolism disorders, and then induce ferroptosis [166]. Acacetin, another flavonoid, has been shown to protect against NAFLD by regulating inflammation and AMPK-related lipid metabolism [167]. A further study showed that acacetin could reduce HFD-induced liver lipid accumulation by inhibiting ER stress-dependent ferroptosis, suggesting that acacetin may be a potential therapeutic drug for NAFLD [168]. In addition, acacetin could also inhibit ER stress and hepatocyte apoptosis by targeting PPARγ, which has a significant protective effect on APAP-induced liver injury [169]. In summary, NAC showed promising therapeutic effect on NAFLD by targeting ferroptosis, and further mechanisms need to be explored.

### 3.4. Liver Fibrosis (LF)

The LF is accompanied by excessive accumulation of extracellular matrix (ECM) proteins, and advanced LF can lead to cirrhosis and liver failure [170]. HSCs are the main type of ECM-secreting cells, and their activation is the core event of LF [171]. Inhibition of HSCs activation, or induction of HSCs death, may be two effective ways to reverse LF [172]. However, the pathogenesis of LF is not fully elucidated, and specific drugs for treating LF do not exist. Increasing evidence suggests that inhibiting hepatocyte ferroptosis or promoting HSCs ferroptosis may be effective ways to treat LF. For example, simvastatin was shown to inhibit the activation of HSCs via triggering ferroptosis [173]. Here, we focused on the NAC targeting ferroptosis for treating LF and analyzed their mechanisms in treating it. Artemisinin, a sesquiterpene lactone drug extracted from the stem and leaf of *Artemisia annua*, has greatly contributed to human resistance to malaria. Recent studies suggested that artemisinin and its derivatives could also fight tumors by inducing ferroptosis, implying their potential in the treatment of LF [174]. Artemether, an artemisinin derivative, was confirmed to induce ferroptosis of HSCs through a P53-dependent mechanism [175]. Further studies showed that artemether reduced the ubiquitination of IRP2 by inhibiting the binding of IRP2 to STIP1 homology and U-box containing protein 1, which increased iron content, and eventually induced ferroptosis of HSCs [176]. Artesunate, a water-soluble hemisuccinate derived from artemisinin, could induce ferroptosis of HSCs by activating ferritinophagy [177]. N6-methyladenosine (m^6^A) is the most abundant modification of eukaryotic mRNA. The m^6^A “reader” proteins YTH domain family 1/2/3 (YTHDF1/2/3) can recognize and direct m^6^A-modified RNA for subsequent processing [178]. Interestingly, dihydroartemisinin (DHA), another artemisinin derivative, could prolong the half-life of BECN1 mRNA through YTHDF1, which in turn promoted ferritinophagy and eventually induced ferroptosis of HSCs [178]. Curcumol, a sesquiterpene extracted from turmeric root, could also promote NOCA4-mediated ferritinophagy and exert an anti-LF effect [179]. In addition, as the main bioactive ingredient of *Rhizoma coptidis*, berberine could modulate ferritin through autophagy/ROS pathway and ubiquitin-proteasome system, which triggered HSCs ferroptosis and inhibited the production of ECM on account of the imbalance of iron homeostasis and the production of ROS [180]. Phlorizin is a flavonoid extracted from the lychee core. High-throughput sequencing of mRNA and lncRNA in liver tissues indicated that phlorizin’s mechanism in treating LF may include ferroptosis, carbon metabolism, and related biomechanical changes [181]. Decursin, an active compound of *Angelicae sinensis radix*, was shown to improve LF [182]. A further study confirmed that decursin could upregulate Fe^2+^ and lipid ROS, and down-regulate GPX4 and GSH in murine HSCs [183]. Celastrol is a bioactive natural triterpenoid extracted from *Tripterygium wilfordii*. Peroxiredoxins (PRDXs), belonging to peroxidases that reduce peroxides, have a conserved cysteine residue as the site of oxidation [184]. A recent study showed that celastrol could directly bind to PRDX1, PRDX2, PRDX4, and PRDX6 via the active cysteine sites and inhibit their antioxidant activities [185]. Moreover, celastrol could upregulate HO-1 activity, leading to excessive heme decomposition and accumulation of Fe^2+^, which eventually induced ferroptosis in activated HSCs [185]. In addition, magnesium isoglycyrrhizinate, a derivative of glycyrrhizinate, also exerted its anti-fibrotic effect by regulating the ferroptosis of HSCs through an HO-1-dependent mechanism [186]. Ellagic acid is a natural polyphenol product isolated from fruits and vegetables. A recent study showed that it exerted its anti-fibrotic activity by enhancing vesicle-associated membrane protein 2 degradation through a proteasome-dependent pathway in HSCs, which resulted in impaired FPN translocation and iron overload [187]. Caveolin-1 (Cav-1) is an integral membrane protein, and its deficiency-mediated ferroptosis plays a significant role in concanavalin A-induced ALI [188]. Isoliquiritigenin (ISL), a flavonoid extracted from the root of *glycyrrhiza uralensis*, showed an anti-inflammatory effect in acute or chronic liver injury models [189]. Interestingly, ISL also promoted ferroptosis of HSCs by promoting Cav-1 expression, which in turn inhibited GPX4 expression and increased TFR1 and DMT1 expression [190]. Ginsenoside Rh2 (G-Rh2), a kind of NAC extracted from ginseng, inhibited HSCs activation through the AKT-mTOR pathway [191]. A further study confirmed that G-Rh2 could also inhibit HSCs activation by enhancing ferroptosis through upregulating interferon regulatory factor 1 to inhibit SLC7A11 [192]. Wild bitter melon (WM), a wild variety of bitter melon, is rich in ethyl acetate, which has strong antioxidant activity [193]. A recent study showed that WM extract treatment could induce overproduction of ROS, activation of ER stress, and ferroptosis in LPS-activated HSC-T6 cells, thereby exerting its anti-fibrotic effect [193]. Chrysophanol, a natural anthraquinone extracted from the rhizomes of *Rheum palmatum*, could also impair hepatitis B virus X protein-induced activation of HSCs through ER stress and GPX4-independent pathways [194]. Lipocalin-2 (LCN2) is a secreted glycoprotein which induces ferroptosis resistance through the transactivation of nucleoprotein 1, which may be the driving force behind ferroptosis resistance [195]. Danshensu, an active molecule extracted from *Salvia miltiorrhiza* herb, was demonstrated to reverse the up-regulation of LCN2 expression induced by LPS in T6 and LX-2 cells, thereby improving LF [195]. Wogonoside (WG), a flavonoid extracted from *Radix baicalensis*, could promote the consumption of SLC7A11, GPX4, and GSH, as well as the production of iron, ROS, and MDA in HSC-T6, but did not affect hepatocytes or macrophages [196]. Importantly, HSCs ferroptosis mediated by the SOCS1/P53/SLC7A11 pathway was associated with the therapeutic effect of WG on LF [196].

In addition to inducing ferroptosis in HSCs, prevention of ferroptosis in hepatocytes also appears to combat LF. Iron overload caused by HO-1 overexpression could cause ferroptosis of hepatocytes, thereby promoting the progression of liver injury and LF [197]. Regarding preventing ferroptosis in hepatocytes, *Mori fructus* aqueous extracts [198] and gandankang formula [199] activated the NRF2 pathway and provided protective effect against LF.

### 3.5. Hepatocellular Carcinoma (HCC)

The HCC, the most common type of liver cancer, is closely related to impaired cell death pathways [200]. Despite advances in treatment, increasing resistance to existing therapies, such as SOR, worsens the prognosis of HCC patients, leading to the search for alternative treatment strategies [201]. The relationship between ferroptosis and HCC is complex: elevated intracellular iron concentration may promote HCC development, and activation of ferroptosis may potentially prevent HCC cell proliferation [202]. Chemotherapy, phytochemicals, nanoparticles, and noncoding RNA have been shown to treat HCC by regulating ferroptosis [203]. SOR, a first-line treatment for HCC, could induce ferroptosis via inhibiting the SLC7A11 or HBXIP/SCD axis in HCC cells [204,205]. Moreover, inhibition of FTH1 renders could sensitize HCC cells to RSL3- and iron-induced ferroptosis [7]. But HCC cells can inhibit ferroptosis through regulatory mechanisms such as the antioxidant regulator NRF2, the transsulfuration pathway, and mechanistic targets of mTOR, thereby continuing tumor growth [203]. Previous studies have shown that NRF2 could enhance drug resistance in HCC through multiple pathways [206,207]. The CBS activation under tumor necrosis factor alpha-induced oxidative stress could also inhibit ferroptosis and promote tumor progression by increasing cystathionine and GSH production in HCC cells [203,208]. Moreover, miR-21-5p could inhibit ferroptosis by regulating the AKT/mTOR pathway in HCC cells [209]. Therefore, it is urgent to develop more drugs targeting ferroptosis for the treatment of HCC. Increasing evidence suggests that NAC can directly trigger ferroptosis in HCC cells or enhance the ferroptosis-inducing ability of anticancer drugs such as SOR to improve the therapeutic effect. DHA was demonstrated to be effective in treating LF, and it also showed great therapeutic potential for liver cancer. Previous studies showed that DHA could trigger ferroptosis of liver cancer cells by activating anti-survival unfolded protein responses, which contributed to increased expression of Chac glutathione specific γ-glutamylcyclotransferase 1 and accelerated formation of phosphatidylethanolamine-binding protein/15-LOX [210,211]. A recent study showed that DHA could strengthen the ability of SOR to trigger ferroptosis in HepG2 cells, as evidenced by lower levels of HO-1, SLC7A11, GSH, GPX4, and recombinant glutamate cysteine ligase, catalytic, as well as increased levels of lipid ROS, LIP, and MDA [212].

Improving drug delivery is one way to make drugs more efficient. For example, nanoscale drug delivery systems (NDDSs) were proven to enhance drug stability and solubility, prolong circulation time, and promote selective accumulation in tumors [213]. Recently, a nanoplatform was constructed by incorporating amphiphilic dendrimers into liposomes for effective co-delivery of the SOR and hemin. The pH-sensitive vesicles could exert potent anticancer potency by inducing ferroptosis and apoptosis in the acidic tumor microenvironment [214]. In addition, a multifunctional nanodrug, which loaded DHA on Fe^3+^-doped MnO_2_ nanosheets (Fe-MnO_2_/DHA), was developed to treat HCC [215]. Fe-MnO_2_/DHA could be degraded to Fe^2+^, Mn^2+^, and DHA by interacting with GSH, which is highly expressed in tumor cells. On the one hand, Fe^2+^, Mn^2+^, and DHA could promote the ferroptosis of tumor cells by producing ROS. On the other hand, Fe-MnO_2_/DHA could mediate the three-pronged stimulation of oxidative stress, which led to high immune activation of immunogenic cell death and polarization of macrophages. Moreover, an angelica polysaccharide-based nanocarrier material encapsulating curcumin in its hydrophobic core was developed to improve water solubility and bioavailability of curcumin and ultimately achieve the dual effects of sensitizing ferroptosis and anti-tumor [216]. Although the mechanism is not identical, artesunate [217], tiliroside [218], metformin [219], ursolic acid [220], camptothecin [221], and withaferin A [222] could also sensitize HCC cells to SOR via inducing ferroptosis (Table 2).

Iron overload is an important cause of ferroptosis. Therefore, NCOA4-mediated ferritinophagy is a significant target of many natural compounds because it may lead to iron overload. Esculetin [223], caryophyllene oxide [224], d-Borneol [225], and electrophilic sesquiterpenes isolated from *Eupatorium chinense* L. [226] could trigger ferroptosis of liver cancer cells through activating NCOA4-mediated ferritinophagy. In addition, SSPH I, a steroidal saponin isolated from *Schizocapsa plantaginea* Hance, could also upregulate the expression of TFR1 and FPN, leading to iron overload and inducing ferroptosis [227].

SLC7A11-GSH-GPX4 axis is the key antioxidant pathway against ferroptosis. Pien-Tze-Huang, a Chinese patent medicine approved by China Food and Drug Administration, may effectively improve the microenvironment of LF and inhibit the occurrence of HCC through triggering ferroptosis of tumor cells via suppressing the SLC7A11-GSH-GPX4 axis [228]. Moreover, rhamnazin [229], polyphyllin VI [230], corosolic acid [231], solasonine [232], parthenolide [233], cryptotanshinone [234], and heteronemin [235] could trigger ferroptosis in HCC cells through down-regulating GPX4 expression. Among them, parthenolide, cryptotanshinone, corosolic acid, and solasonine could also inhibit the synthesis of GSH by rapid thiol oxidation [233] and reducing the expression of SCL7A11 [234] and glutathione synthetase (GSS) [231,232], respectively. Interestingly, aspirin, a derivative of salicylic acid, could trigger ferroptosis by restricting NF-κB-activated SLC7A11 transcription, thereby inhibiting the growth of HCC [236]. ART was shown to treat NAFLD by activating NRF2 and its downstream proteins [154]. However, ART could inhibit the expression of GPX4 and FTL. Meanwhile, it could also upregulate the expression of ACSL4 and TFR1 proteins in HCC cells [237]. Therefore, the regulatory results of ferroptosis by the same natural compound in treating different diseases may be opposite.

As already stated, ER stress can also trigger ferroptosis. Interestingly, eupalinolide B from *Eupatorium lindleyanum* DC could exert an anti-proliferation effect on HCC by activating ferroptosis induced by ER stress and HO-1 activation [238]. Bioinformatics analysis is widely used to explore the complex mechanisms and effective targets of drug treatment. According to the analysis of relevant data, dehydroabietic acid [239], curcumin [240], and *Astragalus membranaceus* [241] could exert their potential effect in treating HCC by regulating ferroptosis-related genes or pathways. It is expected that more effective targets will be identified with the development of various omics techniques and analytical methods.

## 4. Discussion and Prospects

In recent years, the incidence of liver disease has increased due to various factors such as viruses, alcohol, drug abuse, and environmental pollution. NAFLD is one of the most common chronic diseases, and liver cancer has become an important cause of cancer-related death. Liver disease poses a great threat to people’s lives and property. However, effective therapeutic drugs are not still limited due to the pathogenesis of liver disease, such as LF and NAFLD, is still unclear. In addition, the problem of drug resistance in existing treatments for HCC will also remain for a long time. Exploring new pathogenic mechanisms and therapeutic drugs becomes an important task in the current intervention of liver disease. As a new type of RCD, ferroptosis is closely associated with the occurrence and development of various diseases, including liver disease. Here, we summarize the molecular mechanisms of ferroptosis from three aspects: iron metabolism, lipid metabolism, and antioxidant system, which may all be potential therapeutic targets. Several trials have already explored the potential application of ferroptosis regulators such as ferrostatin-1 [85], liproxstatin-1 [146], and erastin [204] in diseases such as liver injury, NASH, and HCC. NAC, including TCM formulas and biological extracts, have the advantages of wide sources, high safety, and multiple therapeutic targets. Mounting evidence shows that NAC have great potential as drugs, nutraceuticals, and even functional foods for treating liver disease associated with ferroptosis [11]. Here, we summarize the NAC experimentally demonstrated to potentially treat liver disease by regulating ferroptosis. These NAC include flavonoids, terpenoids, saponins, esters, alkaloids, organic acids, and so on, and their mechanisms of action are different. Moreover, some TCM formulas, such as gandankang, have also shown good therapeutic effects [102,199].

With the development of biotechnology, such as whole-genome CRISPR activation screen, high-throughput screening, omics technology, and bioinformatics analysis, more therapeutic targets and effective NAC related to ferroptosis will be identified. In addition, modification of drugs such as NDDSs can improve NAC’s solubility, targeting, and permeability, thereby enhancing their bioavailability and potency. Therefore, exploring and modifying NAC targeting ferroptosis has a bright prospect for treating liver disease.

However, some problems still need to be solved in developing and utilizing NAC. NAC are generally less toxic, but this does not guarantee they are all safe. Recent studies have confirmed that triptolide [242], toosendanin [243], aurantio-obtusin [244], and *Epimedium koreanum* Nakai [245] all induced hepatotoxicity by triggering ferroptosis. NAC that triggers ferroptosis in HSCs and HCC cells may also cause damage to healthy hepatocytes. Moreover, the regulatory effects of these compounds on ferroptosis at different stages of disease development are not fully understood. Interestingly, some NAC, such as curcumin and DAA, may exhibit opposite effects on regulating ferroptosis in different cells, causing the disease to progress in a beneficial direction. In addition, some NAC can cause ferroptosis and other types of RCD, such as apoptosis, and it is unclear which one is dominant. Furthermore, the existing studies on NAC for treating liver disease by regulating ferroptosis were mainly carried out on animal or cell in vitro, and molecular mechanisms underlying the therapeutic effects of these NAC require further investigation. For future clinical trials, appropriate drug dosage, efficient delivery methods, and safety evaluation will be the main issues to be addressed [14]. It is also worth exploring whether combining multiple NAC can achieve better efficacy. In conclusion, regulating ferroptosis by NAC is an interesting and promising therapeutic approach due to the wide range of sources and the diversity of mechanisms. Although there are still many problems to be solved in the future, the therapeutic potential of NAC will eventually be realized.

## 5. Conclusions

Liver disease is a worldwide health issue that greatly threatens people’s health. The RCD is widely involved in the occurrence and development of various diseases. As an emerging type of RCD, ferroptosis plays an important role in the pathogenesis of liver disease, including ALI, ALD, NAFLD, LF, and HCC. Therefore, the development of therapies targeting ferroptosis is a promising strategy for the treatment of liver disease. Increasing studies have shown that NAC can improve liver disease by regulating ferroptosis-related signaling pathways, such as NRF2/HO-1. However, the mechanism by which NAC selectively induce ferroptosis in different types of liver cells needs to be further explored. In addition, future research should focus on optimizing drug delivery methods, exploring appropriate drug combinations and doses, and evaluating drug safety. In summary, NAC are potent ferroptosis regulators and are expected to play a great role in the treatment of liver disease.

## Figures and Tables

**Table 1:** Representative NAC as ferroptosis regulators in pollutants-induced liver injury.

Pollutants	Active Compounds	Potential Therapeutic Targets	Ref.
Ammonia	Curcumin	ACSL4, PTGS2, SLC7A1	[131]
Lead	Melatonin	Gut-liver axis, AMPK	[132]
Mercuric chloride	Oleanolic acid	GPX4, SOD1, NRF2, SLC7A11, TFR1	[133]
Ethyl carbamate	Cuttlefish collagen hydrolysate	GSH, HO-1	[134]
Di(2-ethylhexyl) phthalate	Apigenin	System Xc-, GPX4, TFR1, FTH1, FTL, ACSL4, LPCAT3, and PTGS2	[136]
Aflatoxin B1	Total flavonoids of *Rhizoma Drynariae*	GSH, GPX4, Microbiota-Gut-Liver Axis Interaction	[137]
Acrylamide	Quercetin	NCOA4, FTH1	[138]

**Table 2:** Representative NAC as ferroptosis regulators synergizing with SOR.

Active Compounds	Targets	Synergy Mechanism	Ref.
Artesunate	Lysosomal cathepsin B/L activity	Induces oxidative stress and lysosome-mediated ferritinophagy	[217]
Tiliroside	TANK-binding kinase 1 (TBK1)	Promotes KEAP1-mediated NRF2 degradation and inhibits the expression of the downstream target protein of NRF2	[218]
Metformin	ATF4/STAT3	Increases Fe^2+^, ROS, and lipid peroxidation	[219]
Ursolic acid	MCL-1 and SLC7A11	Reduces the synthesis of GSH, increases ROS and lipid peroxidation accumulation	[220]
Camptothecin	NRF2 and SLC7A11.	Increases lipid peroxidation and iron concentration, decreases TAC, GPX4, and GSR activity	[221]
Withaferin A	KEAP1/NRF2	Mitigates NRF2 signaling activation-mediated epithelial to mesenchymal transition (EMT) and SLC7A11 expression.	[222]

## Data Availability

Not applicable.

## References

[1] Galluzzi L., Vitale I., Aaronson S.A., Abrams J.M., Adam D., Agostinis P., Alnemri E.S., Altucci L., Amelio I., Andrews D.W. (2018). Molecular Mechanisms of Cell Death: Recommendations of the Nomenclature Committee on Cell Death 2018. Cell Death Differ..

[2] Tang D., Kang R., Berghe T.V., Vandenabeele P., Kroemer G. (2019). The Molecular Machinery of Regulated Cell Death. Cell Res..

[3] Tang D., Chen X., Kang R., Kroemer G. (2021). Ferroptosis: Molecular Mechanisms and Health Implications. Cell Res..

[4] Dixon S.J., Lemberg K.M., Lamprecht M.R., Skouta R., Zaitsev E.M., Gleason C.E., Patel D.N., Bauer A.J., Cantley A.M., Yang W.S. (2012). Ferroptosis: An Iron-Dependent Form of Non-Apoptotic Cell Death. Cell.

[5] Wang X., Zhou Y., Min J., Wang F. (2023). Zooming in and out of Ferroptosis in Human Disease. Front. Med..

[6] Devarbhavi H., Asrani S.K., Arab J.P., Nartey Y.A., Pose E., Kamath P.S. (2023). Global Burden of Liver Disease: 2023 Update. Hepatology.

[7] Chen J., Li X., Ge C., Min J., Wang F. (2022). The Multifaceted Role of Ferroptosis in Liver Disease. Cell Death Differ..

[8] Ali N., Ferrao K., Mehta K.J. (2023). Liver Iron Loading in Alcohol-Associated Liver Disease. Am. J. Pathol..

[9] Hirako I.C., Antunes M.M., Rezende R.M., Hojo-Souza N.S., Figueiredo M.M., Dias T., Nakaya H., Menezes G.B., Gazzinelli R.T. (2022). Uptake of Plasmodium Chabaudi Hemozoin Drives Kupffer Cell Death and Fuels Superinfections. Sci. Rep..

[10] Kojima H., Hirao H., Kadono K., Ito T., Yao S., Torgerson T., Dery K.J., Kitajima H., Ogawa T., Kaldas F.M. (2024). Cold Stress-Induced Ferroptosis in Liver Sinusoidal Endothelial Cells Determines Liver Transplant Injury and Outcomes. JCI Insight.

[11] Zhang S., Hu R., Geng Y., Chen K., Wang L., Imam M.U. (2021). The Regulatory Effects and the Signaling Pathways of Natural Bioactive Compounds on Ferroptosis. Foods.

[12] Ma N., Zhang Z., Liao F., Jiang T., Tu Y. (2020). The Birth of Artemisinin. Pharmacol. Ther..

[13] Wu Q., Chen Z., Ding Y., Tang Y., Cheng Y. (2022). Protective Effect of Traditional Chinese Medicine on Non-Alcoholic Fatty Liver Disease and Liver Cancer by Targeting Ferroptosis. Front. Nutr..

[14] Zhou Z., Li J., Zhang X. (2023). Natural Flavonoids and Ferroptosis: Potential Therapeutic Opportunities for Human Diseases. J. Agric. Food Chem..

[15] Elmore S. (2007). Apoptosis: A Review of Programmed Cell Death. Toxicol. Pathol..

[16] Torti S.V., Torti F.M. (2013). Iron and Cancer: More Ore to Be Mined. Nat. Rev. Cancer.

[17] Wang F., Lv H., Zhao B., Zhou L., Wang S., Luo J., Liu J., Shang P. (2019). Iron and Leukemia: New Insights for Future Treatments. J. Exp. Clin. Cancer Res. CR.

[18] Wang C.-Y., Babitt J.L. (2019). Liver Iron Sensing and Body Iron Homeostasis. Blood.

[19] Gao Z., Wang D., Zhang H., Yang J., Li M., Lu H., Shen H., Tang Y. (2022). An Iron-Deficient Diet Prevents Alcohol- or Diethylnitrosamine-Induced Acute Hepatotoxicity in Mice by Inhibiting Ferroptosis. Curr. Res. Food Sci..

[20] Arredondo M., Núñez M.T. (2005). Iron and Copper Metabolism. Mol. Aspects Med..

[21] Muckenthaler M.U., Rivella S., Hentze M.W., Galy B. (2017). A Red Carpet for Iron Metabolism. Cell.

[22] Cheng Z., Chu H., Zhu Q., Yang L. (2023). Ferroptosis in Non-Alcoholic Liver Disease: Molecular Mechanisms and Therapeutic Implications. Front. Nutr..

[23] Galaris D., Barbouti A., Pantopoulos K. (2019). Iron Homeostasis and Oxidative Stress: An Intimate Relationship. Biochim. Biophys. Acta BBA -Mol. Cell Res..

[24] Gulec S., Anderson G.J., Collins J.F. (2014). Mechanistic and Regulatory Aspects of Intestinal Iron Absorption. Am. J. Physiol. Gastrointest. Liver Physiol..

[25] Fillebeen C., Gkouvatsos K., Fragoso G., Calvé A., Garcia-Santos D., Buffler M., Becker C., Schümann K., Ponka P., Santos M.M. (2015). Mice Are Poor Heme Absorbers and Do Not Require Intestinal Hmox1 for Dietary Heme Iron Assimilation. Haematologica.

[26] West A.R., Oates P.S. (2008). Mechanisms of Heme Iron Absorption: Current Questions and Controversies. World J. Gastroenterol..

[27] Donovan A., Lima C.A., Pinkus J.L., Pinkus G.S., Zon L.I., Robine S., Andrews N.C. (2005). The Iron Exporter Ferroportin/Slc40a1 Is Essential for Iron Homeostasis. Cell Metab..

[28] Nemeth E., Tuttle M.S., Powelson J., Vaughn M.B., Donovan A., Ward D.M., Ganz T., Kaplan J. (2004). Hepcidin Regulates Cellular Iron Efflux by Binding to Ferroportin and Inducing Its Internalization. Science.

[29] Zheng K., Dong Y., Yang R., Liang Y., Wu H., He Z. (2021). Regulation of Ferroptosis by Bioactive Phytochemicals: Implications for Medical Nutritional Therapy. Pharmacol. Res..

[30] Xiao Y., Xu Z., Cheng Y., Huang R., Xie Y., Tsai H.-I., Zha H., Xi L., Wang K., Cheng X. (2023). Fe3+-Binding Transferrin Nanovesicles Encapsulating Sorafenib Induce Ferroptosis in Hepatocellular Carcinoma. Biomater. Res..

[31] Ohgami R.S., Campagna D.R., Greer E.L., Antiochos B., McDonald A., Chen J., Sharp J.J., Fujiwara Y., Barker J.E., Fleming M.D. (2005). Identification of a Ferrireductase Required for Efficient Transferrin-Dependent Iron Uptake in Erythroid Cells. Nat. Genet..

[32] Fang X., Ardehali H., Min J., Wang F. (2023). The Molecular and Metabolic Landscape of Iron and Ferroptosis in Cardiovascular Disease. Nat. Rev. Cardiol..

[33] Battaglia A.M., Chirillo R., Aversa I., Sacco A., Costanzo F., Biamonte F. (2020). Ferroptosis and Cancer: Mitochondria Meet the “Iron Maiden” Cell Death. Cells.

[34] Yuan X., Li L., Zhang Y., Ai R., Li D., Dou Y., Hou M., Zhao D., Zhao S., Nan Y. (2023). Heme Oxygenase 1 Alleviates Nonalcoholic Steatohepatitis by Suppressing Hepatic Ferroptosis. Lipids Health Dis..

[35] Dang R., Wang M., Li X., Wang H., Liu L., Wu Q., Zhao J., Ji P., Zhong L., Licinio J. (2022). Edaravone Ameliorates Depressive and Anxiety-like Behaviors via Sirt1/Nrf2/HO-1/Gpx4 Pathway. J. Neuroinflamm..

[36] Rouault T.A. (2006). The Role of Iron Regulatory Proteins in Mammalian Iron Homeostasis and Disease. Nat. Chem. Biol..

[37] Bayeva M., Chang H.-C., Wu R., Ardehali H. (2013). When Less Is More: Novel Mechanisms of Iron Conservation. Trends Endocrinol. Metab. TEM.

[38] Bayeva M., Khechaduri A., Puig S., Chang H.-C., Patial S., Blackshear P.J., Ardehali H. (2012). mTOR Regulates Cellular Iron Homeostasis through Tristetraprolin. Cell Metab..

[39] Liang D., Minikes A.M., Jiang X. (2022). Ferroptosis at the Intersection of Lipid Metabolism and Cellular Signaling. Mol. Cell.

[40] Shah R., Shchepinov M.S., Pratt D.A. (2018). Resolving the Role of Lipoxygenases in the Initiation and Execution of Ferroptosis. ACS Cent. Sci..

[41] Nagarajan S.R., Butler L.M., Hoy A.J. (2021). The Diversity and Breadth of Cancer Cell Fatty Acid Metabolism. Cancer Metab..

[42] Krümmel B., von Hanstein A.-S., Plötz T., Lenzen S., Mehmeti I. (2022). Differential Effects of Saturated and Unsaturated Free Fatty Acids on Ferroptosis in Rat β-Cells. J. Nutr. Biochem..

[43] Dierge E., Debock E., Guilbaud C., Corbet C., Mignolet E., Mignard L., Bastien E., Dessy C., Larondelle Y., Feron O. (2021). Peroxidation of N-3 and n-6 Polyunsaturated Fatty Acids in the Acidic Tumor Environment Leads to Ferroptosis-Mediated Anticancer Effects. Cell Metab..

[44] Tesfay L., Paul B.T., Konstorum A., Deng Z., Cox A.O., Lee J., Furdui C.M., Hegde P., Torti F.M., Torti S.V. (2019). Stearoyl-CoA Desaturase 1 Protects Ovarian Cancer Cells from Ferroptotic Cell Death. Cancer Res..

[45] Yamane D., Hayashi Y., Matsumoto M., Nakanishi H., Imagawa H., Kohara M., Lemon S.M., Ichi I. (2022). FADS2-Dependent Fatty Acid Desaturation Dictates Cellular Sensitivity to Ferroptosis and Permissiveness for Hepatitis C Virus Replication. Cell Chem. Biol..

[46] Chen J., Wu K., Lei Y., Huang M., Cheng L., Guan H., Lin J., Zhong M., Wang X., Zheng Z. (2023). Inhibition of Fatty Acid β-Oxidation by Fatty Acid Binding Protein 4 Induces Ferroptosis in HK2 Cells Under High Glucose Conditions. Endocrinol. Metab..

[47] Cao Y., Traer E., Zimmerman G.A., McIntyre T.M., Prescott S.M. (1998). Cloning, Expression, and Chromosomal Localization of Human Long-Chain Fatty Acid-CoA Ligase 4 (FACL4). Genomics.

[48] Li D., Li Y. (2020). The Interaction between Ferroptosis and Lipid Metabolism in Cancer. Signal Transduct. Target. Ther..

[49] Ji Q., Fu S., Zuo H., Huang Y., Chu L., Zhu Y., Hu J., Wu Y., Chen S., Wang Y. (2022). ACSL4 Is Essential for Radiation-Induced Intestinal Injury by Initiating Ferroptosis. Cell Death Discov..

[50] Grube J., Woitok M.M., Mohs A., Erschfeld S., Lynen C., Trautwein C., Otto T. (2022). ACSL4-Dependent Ferroptosis Does Not Represent a Tumor-Suppressive Mechanism but ACSL4 Rather Promotes Liver Cancer Progression. Cell Death Dis..

[51] Reed A., Ichu T.-A., Milosevich N., Melillo B., Schafroth M.A., Otsuka Y., Scampavia L., Spicer T.P., Cravatt B.F. (2022). LPCAT3 Inhibitors Remodel the Polyunsaturated Phospholipid Content of Human Cells and Protect from Ferroptosis. ACS Chem. Biol..

[52] Liang D., Feng Y., Zandkarimi F., Wang H., Zhang Z., Kim J., Cai Y., Gu W., Stockwell B.R., Jiang X. (2023). Ferroptosis Surveillance Independent of GPX4 and Differentially Regulated by Sex Hormones. Cell.

[53] Fan X., Wang X., Hui Y., Zhao T., Mao L., Cui B., Zhong W., Sun C. (2023). Genipin Protects against Acute Liver Injury by Abrogating Ferroptosis via Modification of GPX4 and ALOX15-Launched Lipid Peroxidation in Mice. Apoptosis Int. J. Program. Cell Death.

[54] Rochette L., Dogon G., Rigal E., Zeller M., Cottin Y., Vergely C. (2022). Lipid Peroxidation and Iron Metabolism: Two Corner Stones in the Homeostasis Control of Ferroptosis. Int. J. Mol. Sci..

[55] Brigelius-Flohé R., Maiorino M. (2013). Glutathione Peroxidases. Biochim. Biophys. Acta.

[56] Yang W.S., SriRamaratnam R., Welsch M.E., Shimada K., Skouta R., Viswanathan V.S., Cheah J.H., Clemons P.A., Shamji A.F., Clish C.B. (2014). Regulation of Ferroptotic Cancer Cell Death by GPX4. Cell.

[57] Dixon S.J., Pratt D.A. (2023). Ferroptosis: A Flexible Constellation of Related Biochemical Mechanisms. Mol. Cell.

[58] Yuan S., Wei C., Liu G., Zhang L., Li J., Li L., Cai S., Fang L. (2022). Sorafenib Attenuates Liver Fibrosis by Triggering Hepatic Stellate Cell Ferroptosis via HIF-1α/SLC7A11 Pathway. Cell Prolif..

[59] Liu Y., Wan Y., Jiang Y., Zhang L., Cheng W. (2023). GPX4: The Hub of Lipid Oxidation, Ferroptosis, Disease and Treatment. Biochim. Biophys. Acta BBA -Rev. Cancer.

[60] Ingold K.U., Pratt D.A. (2014). Advances in Radical-Trapping Antioxidant Chemistry in the 21st Century: A Kinetics and Mechanisms Perspective. Chem. Rev..

[61] Bersuker K., Hendricks J.M., Li Z., Magtanong L., Ford B., Tang P.H., Roberts M.A., Tong B., Maimone T.J., Zoncu R. (2019). The CoQ Oxidoreductase FSP1 Acts Parallel to GPX4 to Inhibit Ferroptosis. Nature.

[62] Doll S., Freitas F.P., Shah R., Aldrovandi M., da Silva M.C., Ingold I., Goya Grocin A., Xavier da Silva T.N., Panzilius E., Scheel C.H. (2019). FSP1 Is a Glutathione-Independent Ferroptosis Suppressor. Nature.

[63] Mishima E., Ito J., Wu Z., Nakamura T., Wahida A., Doll S., Tonnus W., Nepachalovich P., Eggenhofer E., Aldrovandi M. (2022). A Non-Canonical Vitamin K Cycle Is a Potent Ferroptosis Suppressor. Nature.

[64] Nakamura T., Hipp C., Santos Dias Mourão A., Borggräfe J., Aldrovandi M., Henkelmann B., Wanninger J., Mishima E., Lytton E., Emler D. (2023). Phase Separation of FSP1 Promotes Ferroptosis. Nature.

[65] Werner E.R., Blau N., Thöny B. (2011). Tetrahydrobiopterin: Biochemistry and Pathophysiology. Biochem. J..

[66] Kraft V.A.N., Bezjian C.T., Pfeiffer S., Ringelstetter L., Müller C., Zandkarimi F., Merl-Pham J., Bao X., Anastasov N., Kössl J. (2020). GTP Cyclohydrolase 1/Tetrahydrobiopterin Counteract Ferroptosis through Lipid Remodeling. ACS Cent. Sci..

[67] Mao C., Liu X., Zhang Y., Lei G., Yan Y., Lee H., Koppula P., Wu S., Zhuang L., Fang B. (2021). DHODH-Mediated Ferroptosis Defence Is a Targetable Vulnerability in Cancer. Nature.

[68] Zi L., Ma W., Zhang L., Qiao B., Qiu Z., Xu J., Zhang J., Ye Y., Yang Y., Dong K. (2023). Uridine Inhibits Hepatocellular Carcinoma Cell Development by Inducing Ferroptosis. J. Clin. Med..

[69] Villa E., Ali E.S., Sahu U., Ben-Sahra I. (2019). Cancer Cells Tune the Signaling Pathways to Empower de Novo Synthesis of Nucleotides. Cancers.

[70] Mishima E., Nakamura T., Zheng J., Zhang W., Mourão A.S.D., Sennhenn P., Conrad M. (2023). DHODH Inhibitors Sensitize to Ferroptosis by FSP1 Inhibition. Nature.

[71] Ida T., Sawa T., Ihara H., Tsuchiya Y., Watanabe Y., Kumagai Y., Suematsu M., Motohashi H., Fujii S., Matsunaga T. (2014). Reactive Cysteine Persulfides and S-Polythiolation Regulate Oxidative Stress and Redox Signaling. Proc. Natl. Acad. Sci. USA.

[72] Ezeriņa D., Takano Y., Hanaoka K., Urano Y., Dick T.P. (2018). N-Acetyl Cysteine Functions as a Fast-Acting Antioxidant by Triggering Intracellular H2S and Sulfane Sulfur Production. Cell Chem. Biol..

[73] Barayeu U., Schilling D., Eid M., Xavier da Silva T.N., Schlicker L., Mitreska N., Zapp C., Gräter F., Miller A.K., Kappl R. (2023). Hydropersulfides Inhibit Lipid Peroxidation and Ferroptosis by Scavenging Radicals. Nat. Chem. Biol..

[74] Wu Z., Barayeu U., Schilling D., Dick T.P., Pratt D.A. (2023). Emergence of (Hydro)Persulfides as Suppressors of Lipid Peroxidation and Ferroptotic Cell Death. Curr. Opin. Chem. Biol..

[75] Erdélyi K., Ditrói T., Johansson H.J., Czikora Á., Balog N., Silwal-Pandit L., Ida T., Olasz J., Hajdú D., Mátrai Z. (2021). Reprogrammed Transsulfuration Promotes Basal-like Breast Tumor Progression via Realigning Cellular Cysteine Persulfidation. Proc. Natl. Acad. Sci. USA.

[76] Song Y.-H., Lei H.-X., Yu D., Zhu H., Hao M.-Z., Cui R.-H., Meng X.-S., Sheng X.-H., Zhang L. (2023). Endogenous Chemicals Guard Health through Inhibiting Ferroptotic Cell Death. BioFactors.

[77] Kapralov A.A., Yang Q., Dar H.H., Tyurina Y.Y., Anthonymuthu T.S., Kim R., St Croix C.M., Mikulska-Ruminska K., Liu B., Shrivastava I.H. (2020). Redox Lipid Reprogramming Commands Susceptibility of Macrophages and Microglia to Ferroptotic Death. Nat. Chem. Biol..

[78] Chen S., Zhu J.-Y., Zang X., Zhai Y.-Z. (2021). The Emerging Role of Ferroptosis in Liver Diseases. Front. Cell Dev. Biol..

[79] Capelletti M.M., Manceau H., Puy H., Peoc’h K. (2020). Ferroptosis in Liver Diseases: An Overview. Int. J. Mol. Sci..

[80] Wu J., Wang Y., Jiang R., Xue R., Yin X., Wu M., Meng Q. (2021). Ferroptosis in Liver Disease: New Insights into Disease Mechanisms. Cell Death Discov..

[81] Stravitz R.T., Fontana R.J., Karvellas C., Durkalski V., McGuire B., Rule J.A., Tujios S., Lee W.M. (2023). Acute Liver Failure Study Group. Future Directions in Acute Liver Failure. Hepatology.

[82] Grek A., Arasi L. (2016). Acute Liver Failure. AACN Adv. Crit. Care.

[83] Liu J., Huang C., Liu J., Meng C., Gu Q., Du X., Yan M., Yu Y., Liu F., Xia C. (2023). Nrf2 and Its Dependent Autophagy Activation Cooperatively Counteract Ferroptosis to Alleviate Acute Liver Injury. Pharmacol. Res..

[84] Yamada N., Karasawa T., Kimura H., Watanabe S., Komada T., Kamata R., Sampilvanjil A., Ito J., Nakagawa K., Kuwata H. (2020). Ferroptosis Driven by Radical Oxidation of N-6 Polyunsaturated Fatty Acids Mediates Acetaminophen-Induced Acute Liver Failure. Cell Death Dis..

[85] Lőrincz T., Jemnitz K., Kardon T., Mandl J., Szarka A. (2015). Ferroptosis Is Involved in Acetaminophen Induced Cell Death. Pathol. Oncol. Res. POR.

[86] Dodson M., Castro-Portuguez R., Zhang D.D. (2019). NRF2 Plays a Critical Role in Mitigating Lipid Peroxidation and Ferroptosis. Redox Biol..

[87] Wang Y.-Q., Wei J.-G., Tu M.-J., Gu J.-G., Zhang W. (2018). Fucoidan Alleviates Acetaminophen-Induced Hepatotoxicity via Oxidative Stress Inhibition and Nrf2 Translocation. Int. J. Mol. Sci..

[88] An Y., Luo Q., Han D., Guan L. (2023). Abietic Acid Inhibits Acetaminophen-Induced Liver Injury by Alleviating Inflammation and Ferroptosis through Regulating Nrf2/HO-1 Axis. Int. Immunopharmacol..

[89] Cai X., Hua S., Deng J., Du Z., Zhang D., Liu Z., Khan N.U., Zhou M., Chen Z. (2022). Astaxanthin Activated the Nrf2/HO-1 Pathway to Enhance Autophagy and Inhibit Ferroptosis, Ameliorating Acetaminophen-Induced Liver Injury. ACS Appl. Mater. Interfaces.

[90] Wang M., Liu C.-Y., Wang T., Yu H.-M., Ouyang S.-H., Wu Y.-P., Gong H.-B., Ma X.-H., Jiao G.-L., Fu L.-L. (2020). (+)-Clausenamide Protects against Drug-Induced Liver Injury by Inhibiting Hepatocyte Ferroptosis. Cell Death Dis..

[91] Zeng Y., Wu R., Wang F., Li S., Li L., Li Y., Qin P., Wei M., Yang J., Wu J. (2023). Liberation of Daidzein by Gut Microbial β-Galactosidase Suppresses Acetaminophen-Induced Hepatotoxicity in Mice. Cell Host Microbe.

[92] Liu T., Yang L., Gao H., Zhuo Y., Tu Z., Wang Y., Xun J., Zhang Q., Zhang L., Wang X. (2022). 3,4-Dihydroxyphenylethyl Alcohol Glycoside Reduces Acetaminophen-Induced Acute Liver Failure in Mice by Inhibiting Hepatocyte Ferroptosis and Pyroptosis. PeerJ.

[93] He Y., Liu C.-Y., He C.-C., Zhao J., Sun Y.-H., Xu H.-S., Cai X.-Q., Li Y.-F., Hiroshi K., He R.-R. (2018). [Protective effect of Fuzheng Yanggan Mixture on drug-induced liver injury]. Zhongguo Zhong Yao Za Zhi Zhongguo Zhongyao Zazhi China J. Chin. Mater. Medica.

[94] Li J., Lu Q., Peng M., Liao J., Zhang B., Yang D., Huang P., Yang Y., Zhao Q., Han B. (2023). Water Extract from Herpetospermum Pedunculosum Attenuates Oxidative Stress and Ferroptosis Induced by Acetaminophen via Regulating Nrf2 and NF-κB Pathways. J. Ethnopharmacol..

[95] Li X., Ma N., Xu J., Zhang Y., Yang P., Su X., Xing Y., An N., Yang F., Zhang G. (2021). Targeting Ferroptosis: Pathological Mechanism and Treatment of Ischemia-Reperfusion Injury. Oxid. Med. Cell. Longev..

[96] Xu J., Li Y., Kang M., Chang C., Wei H., Zhang C., Chen Y. (2023). Multiple Forms of Cell Death: A Focus on the PI3K/AKT Pathway. J. Cell. Physiol..

[97] Ma X., Xu J., Gao N., Tian J., Song T. (2023). Dexmedetomidine Attenuates Myocardial Ischemia-Reperfusion Injury via Inhibiting Ferroptosis by the cAMP/PKA/CREB Pathway. Mol. Cell. Probes.

[98] Chen K., Xue R., Geng Y., Zhang S. (2022). Galangin Inhibited Ferroptosis through Activation of the PI3K/AKT Pathway in Vitro and in Vivo. FASEB J. Off. Publ. Fed. Am. Soc. Exp. Biol..

[99] Qiu S., Li X., Zhang J., Shi P., Cao Y., Zhuang Y., Tong L. (2023). Neutrophil Membrane-Coated Taurine Nanoparticles Protect against Hepatic Ischemia-Reperfusion Injury. Eur. J. Pharmacol..

[100] Li Y., Yu P., Fu W., Wang S., Zhao W., Ma Y., Wu Y., Cui H., Yu X., Fu L. (2023). Ginsenoside Rd Inhibited Ferroptosis to Alleviate CCl4-Induced Acute Liver Injury in Mice via cGAS/STING Pathway. Am. J. Chin. Med..

[101] Zhao T., Yu Z., Zhou L., Wang X., Hui Y., Mao L., Fan X., Wang B., Zhao X., Sun C. (2022). Regulating Nrf2-GPx4 Axis by Bicyclol Can Prevent Ferroptosis in Carbon Tetrachloride-Induced Acute Liver Injury in Mice. Cell Death Discov..

[102] Wei Y.-Y., Wang H.-R., Fan Y.-M., Gu J.-H., Zhang X.-Y., Gong X.-H., Hao Z.-H. (2023). Acute Liver Injury Induced by Carbon Tetrachloride Reversal by Gandankang Aqueous Extracts through Nuclear Factor Erythroid 2-Related Factor 2 Signaling Pathway. Ecotoxicol. Environ. Saf..

[103] Dai C., Li H., Wang Y., Tang S., Velkov T., Shen J. (2021). Inhibition of Oxidative Stress and ALOX12 and NF-κB Pathways Contribute to the Protective Effect of Baicalein on Carbon Tetrachloride-Induced Acute Liver Injury. Antioxidants.

[104] Li J., Huang Q., Lv M., Ma W., Sun J., Zhong X., Hu R., Ma M., Han Z., Zhang W. (2023). Role of Liensinine in Sensitivity of Activated Macrophages to Ferroptosis and in Acute Liver Injury. Cell Death Discov..

[105] Zhao C., Xiao C., Feng S., Bai J. (2023). Artemisitene Alters LPS-Induced Oxidative Stress, Inflammation and Ferroptosis in Liver Through Nrf2/HO-1 and NF-kB Pathway. Front. Pharmacol..

[106] Wang Y., Chen Q., Shi C., Jiao F., Gong Z. (2019). Mechanism of Glycyrrhizin on Ferroptosis during Acute Liver Failure by Inhibiting Oxidative Stress. Mol. Med. Rep..

[107] Ji Y., Si W., Zeng J., Huang L., Huang Z., Zhao L., Liu J., Zhu M., Kuang W. (2021). Niujiaodihuang Detoxify Decoction Inhibits Ferroptosis by Enhancing Glutathione Synthesis in Acute Liver Failure Models. J. Ethnopharmacol..

[108] Dai W., Pang X., Peng W., Zhan X., Chen C., Zhao W., Zeng C., Mei Q., Chen Q., Kuang W. (2023). Liver Protection of a Low-Polarity Fraction from Ficus Pandurata Hance, Prepared by Supercritical CO2 Fluid Extraction, on CCl4-Induced Acute Liver Injury in Mice via Inhibiting Apoptosis and Ferroptosis Mediated by Strengthened Antioxidation. Molecules.

[109] Samson N., Ablasser A. (2022). The cGAS–STING Pathway and Cancer. Nat. Cancer.

[110] Qiu S., Zhong X., Meng X., Li S., Qian X., Lu H., Cai J., Zhang Y., Wang M., Ye Z. (2023). Mitochondria-Localized cGAS Suppresses Ferroptosis to Promote Cancer Progression. Cell Res..

[111] Siregar A.S., Nyiramana M.M., Kim E.-J., Cho S.B., Woo M.S., Lee D.K., Hong S.-G., Han J., Kang S.S., Kim D.R. (2021). Oyster-Derived Tyr-Ala (YA) Peptide Prevents Lipopolysaccharide/D-Galactosamine-Induced Acute Liver Failure by Suppressing Inflammatory, Apoptotic, Ferroptotic, and Pyroptotic Signals. Mar. Drugs.

[112] Macías-Rodríguez R.U., Inzaugarat M.E., Ruiz-Margáin A., Nelson L.J., Trautwein C., Cubero F.J. (2020). Reclassifying Hepatic Cell Death during Liver Damage: Ferroptosis—A Novel Form of Non-Apoptotic Cell Death?. Int. J. Mol. Sci..

[113] Shi J.-F., Liu Y., Wang Y., Gao R., Wang Y., Liu J. (2023). Targeting Ferroptosis, a Novel Programmed Cell Death, for the Potential of Alcohol-Related Liver Disease Therapy. Front. Pharmacol..

[114] Ding Q., Pi A., Hao L., Xu T., Zhu Q., Shu L., Yu X., Wang W., Si C., Li S. (2023). Genistein Protects against Acetaldehyde-Induced Oxidative Stress and Hepatocyte Injury in Chronic Alcohol-Fed Mice. J. Agric. Food Chem..

[115] Yang C., Wu A., Tan L., Tang D., Chen W., Lai X., Gu K., Chen J., Chen D., Tang Q. (2023). Epigallocatechin-3-Gallate Alleviates Liver Oxidative Damage Caused by Iron Overload in Mice through Inhibiting Ferroptosis. Nutrients.

[116] El Rashed Z., Lupidi G., Kanaan H., Grasselli E., Canesi L., Khalifeh H., Demori I. (2021). Antioxidant and Antisteatotic Activities of a New Fucoidan Extracted from Ferula Hermonis Roots Harvested on Lebanese Mountains. Molecules.

[117] Xue M., Tian Y., Sui Y., Zhao H., Gao H., Liang H., Qiu X., Sun Z., Zhang Y., Qin Y. (2022). Protective Effect of Fucoidan against Iron Overload and Ferroptosis-Induced Liver Injury in Rats Exposed to Alcohol. Biomed. Pharmacother..

[118] Song X.-Y., Liu P.-C., Liu W.-W., Zhou J., Hayashi T., Mizuno K., Hattori S., Fujisaki H., Ikejima T. (2022). Silibinin Inhibits Ethanol- or Acetaldehyde-Induced Ferroptosis in Liver Cell Lines. Toxicol. Vitro Int. J. Publ. Assoc. BIBRA.

[119] Eiyama A., Okamoto K. (2015). PINK1/Parkin-Mediated Mitophagy in Mammalian Cells. Curr. Opin. Cell Biol..

[120] Dong J., Du C., Xu C., Wang Q., Wang Z., Zhu Q., Lv X., Zhang L., Li J., Huang C. (2023). Verbenalin Attenuates Hepatic Damage and Mitochondrial Dysfunction in Alcohol-Associated Steatohepatitis by Regulating MDMX/PPARα-Mediated Ferroptosis. J. Ethnopharmacol..

[121] Fang C., Zhang J., Han J., Lei Y., Cao Z., Pan J., Pan Z., Zhang Z., Qu N., Luo H. (2023). Tiaogan Jiejiu Tongluo Formula Attenuated Alcohol-Induced Chronic Liver Injury by Regulating Lipid Metabolism in Rats. J. Ethnopharmacol..

[122] Liu J., Yang M., Kang R., Klionsky D.J., Tang D. (2019). Autophagic Degradation of the Circadian Clock Regulator Promotes Ferroptosis. Autophagy.

[123] Zhao Y., Zhang R., Wang Z., Chen Z., Wang G., Guan S., Lu J. (2022). Melatonin Prevents against Ethanol-Induced Liver Injury by Mitigating Ferroptosis via Targeting Brain and Muscle ARNT-like 1 in Mice Liver and HepG2 Cells. J. Agric. Food Chem..

[124] He P., Hua H., Tian W., Zhu H., Liu Y., Xu X. (2020). Holly (Ilex Latifolia Thunb.) Polyphenols Extracts Alleviate Hepatic Damage by Regulating Ferroptosis Following Diquat Challenge in a Piglet Model. Front. Nutr..

[125] Hua H., Xu X., Tian W., Li P., Zhu H., Wang W., Liu Y., Xiao K. (2022). Glycine Alleviated Diquat-Induced Hepatic Injury via Inhibiting Ferroptosis in Weaned Piglets. Anim. Biosci..

[126] Cui W., Zhou H., Zhang J., Zhang J., Wu D., Rong Y., Liu F., Liu J., Liu H., Wei B. (2023). Hepatoprotective Effect of Artemisia Argyi Essential Oil on Bisphenol A-Induced Hepatotoxicity via Inhibition of Ferroptosis in Mice. Environ. Toxicol..

[127] Mahlooji M.A., Heshmati A., Kheiripour N., Ghasemi H., Asl S.S., Solgi G., Ranjbar A., Hosseini A. (2022). Evaluation of Protective Effects of Curcumin and Nanocurcumin on Aluminium Phosphide-Induced Subacute Lung Injury in Rats: Modulation of Oxidative Stress through SIRT1/FOXO3 Signalling Pathway. Drug Res..

[128] Wang D., Yin K., Zhang Y., Lu H., Hou L., Zhao H., Xing M. (2023). Novel Pathways of Fluoride-Induced Hepatotoxicity: P53-Dependent Ferroptosis Induced by the SIRT1/FOXOs Pathway and Nrf2/HO-1 Pathway. Comp. Biochem. Physiol. Toxicol. Pharmacol. CBP.

[129] Zhao Y., Liu X., Liang C., Pei T., Guo M., Wang J., Zhang J. (2022). α-Lipoic Acid Alleviated Fluoride-Induced Hepatocyte Injury via Inhibiting Ferroptosis. J. Agric. Food Chem..

[130] Liu Y., Zhu W., Ni D., Zhou Z., Gu J.-H., Zhang W., Sun H., Liu F. (2020). Alpha Lipoic Acid Antagonizes Cytotoxicity of Cobalt Nanoparticles by Inhibiting Ferroptosis-like Cell Death. J. Nanobiotechnol..

[131] Wu L., Dong B., Chen Q., Wang Y., Han D., Zhu X., Liu H., Zhang Z., Yang Y., Xie S. (2023). Effects of Curcumin on Oxidative Stress and Ferroptosis in Acute Ammonia Stress-Induced Liver Injury in Gibel Carp (*Carassius Gibelio*). Int. J. Mol. Sci..

[132] Miao Z., Miao Z., Teng X., Xu S. (2023). Melatonin Alleviates Lead-Induced Fatty Liver in the Common Carps (Cyprinus Carpio) via Gut-Liver Axis. Environ. Pollut. Barking Essex 1987.

[133] Ouyang C., Ma X., Zhao J., Li S., Liu C., Tang Y., Zhou J., Chen J., Li X., Li W. (2023). Oleanolic Acid Inhibits Mercury Chloride Induced-Liver Ferroptosis by Regulating ROS/Iron Overload. Ecotoxicol. Environ. Saf..

[134] Du B., Deng G., Zaman F., Ma H., Li X., Chen J., Li T., Huang Y. (2021). Antioxidant Cuttlefish Collagen Hydrolysate against Ethyl Carbamate-Induced Oxidative Damage. RSC Adv..

[135] Xu Y., Li Y., Li J., Chen W. (2022). Ethyl Carbamate Triggers Ferroptosis in Liver through Inhibiting GSH Synthesis and Suppressing Nrf2 Activation. Redox Biol..

[136] Han D., Yao Y., Chen L., Miao Z., Xu S. (2022). Apigenin Ameliorates Di(2-Ethylhexyl) Phthalate-Induced Ferroptosis: The Activation of Glutathione Peroxidase 4 and Suppression of Iron Intake. Food Chem. Toxicol. Int. J. Publ. Br. Ind. Biol. Res. Assoc..

[137] Huang S., Lin L., Wang S., Ding W., Zhang C., Shaukat A., Xu B., Yue K., Zhang C., Liu F. (2023). Total Flavonoids of Rhizoma Drynariae Mitigates Aflatoxin B1-Induced Liver Toxicity in Chickens via Microbiota-Gut-Liver Axis Interaction Mechanisms. Antioxidants.

[138] Huang T., Zhang K., Wang J., He K., Zhou X., Nie S. (2023). Quercetin Alleviates Acrylamide-Induced Liver Injury by Inhibiting Autophagy-Dependent Ferroptosis. J. Agric. Food Chem..

[139] Han S.K., Baik S.K., Kim M.Y. (2023). Non-Alcoholic Fatty Liver Disease: Definition and Subtypes. Clin. Mol. Hepatol..

[140] Ji J., Wu L., Wei J., Wu J., Guo C. (2023). The Gut Microbiome and Ferroptosis in MAFLD. J. Clin. Transl. Hepatol..

[141] Zhao J., Hu Y., Peng J. (2021). Targeting Programmed Cell Death in Metabolic Dysfunction-Associated Fatty Liver Disease (MAFLD): A Promising New Therapy. Cell. Mol. Biol. Lett..

[142] Zhao S., Guo Y., Yin X. (2024). Autophagy, Ferroptosis, Apoptosis and Pyroptosis in Metabolic Dysfunction-Associated Steatotic Liver Disease. Front. Biosci. Landmark Ed..

[143] Pierantonelli I., Svegliati-Baroni G. (2019). Nonalcoholic Fatty Liver Disease: Basic Pathogenetic Mechanisms in the Progression From NAFLD to NASH. Transplantation.

[144] Loguercio C., De Girolamo V., de Sio I., Tuccillo C., Ascione A., Baldi F., Budillon G., Cimino L., Di Carlo A., Di Marino M.P. (2001). Non-Alcoholic Fatty Liver Disease in an Area of Southern Italy: Main Clinical, Histological, and Pathophysiological Aspects. Hepatology.

[145] Ma C., Han L., Zhu Z., Heng Pang C., Pan G. (2022). Mineral Metabolism and Ferroptosis in Non-Alcoholic Fatty Liver Diseases. Biochem. Pharmacol..

[146] Qi J., Kim J.-W., Zhou Z., Lim C.-W., Kim B. (2020). Ferroptosis Affects the Progression of Nonalcoholic Steatohepatitis via the Modulation of Lipid Peroxidation-Mediated Cell Death in Mice. Am. J. Pathol..

[147] Qiu M., Xiao F., Wang T., Piao S., Zhao W., Shao S., Yan M., Zhao D. (2020). Protective Effect of Hedansanqi Tiaozhi Tang against Non-Alcoholic Fatty Liver Disease in Vitro and in Vivo through Activating Nrf2/HO-1 Antioxidant Signaling Pathway. Phytomedicine.

[148] Xu Q., Fan Y., Loor J.J., Liang Y., Lv H., Sun X., Jia H., Xu C. (2021). Aloin Protects Mice from Diet-Induced Non-Alcoholic Steatohepatitis via Activation of Nrf2/HO-1 Signaling. Food Funct..

[149] Kim W.-J., Kim W., Bae J.-M., Gim J., Kim S.-J. (2021). Dehydroabietic Acid Is a Novel Survivin Inhibitor for Gastric Cancer. Plants.

[150] Kim E., Kang Y.-G., Kim Y.-J., Lee T.R., Yoo B.C., Jo M., Kim J.H., Kim J.-H., Kim D., Cho J.Y. (2019). Dehydroabietic Acid Suppresses Inflammatory Response Via Suppression of Src-, Syk-, and TAK1-Mediated Pathways. Int. J. Mol. Sci..

[151] da Silva K.R., Damasceno J.L., Inácio M.d.O., Abrão F., Ferreira N.H., Tavares D.C., Ambrosio S.R., Veneziani R.C.S., Martins C.H.G. (2019). Antibacterial and Cytotoxic Activities of Pinus Tropicalis and Pinus Elliottii Resins and of the Diterpene Dehydroabietic Acid Against Bacteria That Cause Dental Caries. Front. Microbiol..

[152] Gao G., Xie Z., Li E.-W., Yuan Y., Fu Y., Wang P., Zhang X., Qiao Y., Xu J., Hölscher C. (2021). Dehydroabietic Acid Improves Nonalcoholic Fatty Liver Disease through Activating the Keap1/Nrf2-ARE Signaling Pathway to Reduce Ferroptosis. J. Nat. Med..

[153] Xie Z., Gao G., Wang H., Li E., Yuan Y., Xu J., Zhang Z., Wang P., Fu Y., Zeng H. (2020). Dehydroabietic Acid Alleviates High Fat Diet-Induced Insulin Resistance and Hepatic Steatosis through Dual Activation of PPAR-γ and PPAR-α. Biomed. Pharmacother..

[154] Ye Q., Jiang Y., Wu D., Cai J., Jiang Z., Zhou Z., Liu L., Ling Q., Wang Q., Zhao G. (2023). Atractylodin Alleviates Nonalcoholic Fatty Liver Disease by Regulating Nrf2-Mediated Ferroptosis. Heliyon.

[155] Yang Y., Chen J., Gao Q., Shan X., Wang J., Lv Z. (2020). Study on the Attenuated Effect of Ginkgolide B on Ferroptosis in High Fat Diet Induced Nonalcoholic Fatty Liver Disease. Toxicology.

[156] Xu J., Tian H., Ji Y., Dong L., Liu Y., Wang Y., Gao X., Shi H., Li H., Yang L. (2023). Urolithin C Reveals Anti-NAFLD Potential via AMPK-Ferroptosis Axis and Modulating Gut Microbiota. Naunyn. Schmiedebergs Arch. Pharmacol..

[157] Castillo V., Figueroa F., González-Pizarro K., Jopia P., Ibacache-Quiroga C. (2021). Probiotics and Prebiotics as a Strategy for Non-Alcoholic Fatty Liver Disease, a Narrative Review. Foods.

[158] Jiang L., Hickman J.H., Wang S.-J., Gu W. (2015). Dynamic Roles of P53-Mediated Metabolic Activities in ROS-Induced Stress Responses. Cell Cycle Georget. Tex.

[159] Ou Y., Wang S.-J., Li D., Chu B., Gu W. (2016). Activation of SAT1 Engages Polyamine Metabolism with P53-Mediated Ferroptotic Responses. Proc. Natl. Acad. Sci. USA.

[160] Liu H., Yan J., Guan F., Jin Z., Xie J., Wang C., Liu M., Liu J. (2023). Zeaxanthin Prevents Ferroptosis by Promoting Mitochondrial Function and Inhibiting the P53 Pathway in Free Fatty Acid-Induced HepG2 Cells. Biochim. Biophys. Acta Mol. Cell Biol. Lipids.

[161] Moore M.P., Cunningham R.P., Meers G.M., Johnson S.A., Wheeler A.A., Ganga R.R., Spencer N.M., Pitt J.B., Diaz-Arias A., Swi A.I.A. (2022). Compromised Hepatic Mitochondrial Fatty Acid Oxidation and Reduced Markers of Mitochondrial Turnover in Human NAFLD. Hepatology.

[162] Shum M., Ngo J., Shirihai O.S., Liesa M. (2021). Mitochondrial Oxidative Function in NAFLD: Friend or Foe?. Mol. Metab..

[163] Simões I.C.M., Fontes A., Pinton P., Zischka H., Wieckowski M.R. (2018). Mitochondria in Non-Alcoholic Fatty Liver Disease. Int. J. Biochem. Cell Biol..

[164] Ding S.-B., Chu X.-L., Jin Y.-X., Jiang J.-J., Zhao X., Yu M. (2023). Epigallocatechin Gallate Alleviates High-Fat Diet-Induced Hepatic Lipotoxicity by Targeting Mitochondrial ROS-Mediated Ferroptosis. Front. Pharmacol..

[165] Jiang J.-J., Zhang G.-F., Zheng J.-Y., Sun J.-H., Ding S.-B. (2022). Targeting Mitochondrial ROS-Mediated Ferroptosis by Quercetin Alleviates High-Fat Diet-Induced Hepatic Lipotoxicity. Front. Pharmacol..

[166] Wu C., Du M., Yu R., Cheng Y., Wu B., Fu J., Tan W., Zhou Q., Balawi E., Liao Z.B. (2022). A Novel Mechanism Linking Ferroptosis and Endoplasmic Reticulum Stress via the circPtpn14/miR-351-5p/5-LOX Signaling in Melatonin-Mediated Treatment of Traumatic Brain Injury. Free Radic. Biol. Med..

[167] Liou C.-J., Wu S.-J., Shen S.-C., Chen L.-C., Chen Y.-L., Huang W.-C. (2022). Acacetin Protects against Non-Alcoholic Fatty Liver Disease by Regulating Lipid Accumulation and Inflammation in Mice. Int. J. Mol. Sci..

[168] Jiang Z., Sun H., Miao J., Sheng Q., Xu J., Gao Z., Zhang X., Song Y., Chen K. (2023). The Natural Flavone Acacetin Protects against High-Fat Diet-Induced Lipid Accumulation in the Liver via the Endoplasmic Reticulum Stress/Ferroptosis Pathway. Biochem. Biophys. Res. Commun..

[169] Miao J., Yao S., Sun H., Jiang Z., Gao Z., Xu J., Chen K. (2023). Protective Effect of Water-Soluble Acacetin Prodrug on APAP-Induced Acute Liver Injury Is Associated with Upregulation of PPARγ and Alleviation of ER Stress. Int. J. Mol. Sci..

[170] Bataller R., Brenner D.A. (2005). Liver Fibrosis. J. Clin. Invest..

[171] Aydın M.M., Akçalı K.C. (2018). Liver Fibrosis. Turk. J. Gastroenterol. Off. J. Turk. Soc. Gastroenterol..

[172] Pei Q., Yi Q., Tang L. (2023). Liver Fibrosis Resolution: From Molecular Mechanisms to Therapeutic Opportunities. Int. J. Mol. Sci..

[173] Kitsugi K., Noritake H., Matsumoto M., Hanaoka T., Umemura M., Yamashita M., Takatori S., Ito J., Ohta K., Chida T. (2023). Simvastatin Inhibits Hepatic Stellate Cells Activation by Regulating the Ferroptosis Signaling Pathway. Biochim. Biophys. Acta Mol. Basis Dis..

[174] Hu Y., Guo N., Yang T., Yan J., Wang W., Li X. (2022). The Potential Mechanisms by Which Artemisinin and Its Derivatives Induce Ferroptosis in the Treatment of Cancer. Oxid. Med. Cell. Longev..

[175] Wang L., Zhang Z., Li M., Wang F., Jia Y., Zhang F., Shao J., Chen A., Zheng S. (2019). P53-Dependent Induction of Ferroptosis Is Required for Artemether to Alleviate Carbon Tetrachloride-Induced Liver Fibrosis and Hepatic Stellate Cell Activation. IUBMB Life.

[176] Li Y., Jin C., Shen M., Wang Z., Tan S., Chen A., Wang S., Shao J., Zhang F., Zhang Z. (2020). Iron Regulatory Protein 2 Is Required for Artemether -Mediated Anti-Hepatic Fibrosis through Ferroptosis Pathway. Free Radic. Biol. Med..

[177] Kong Z., Liu R., Cheng Y. (2019). Artesunate Alleviates Liver Fibrosis by Regulating Ferroptosis Signaling Pathway. Biomed. Pharmacother..

[178] Shen M., Guo M., Li Y., Wang Y., Qiu Y., Shao J., Zhang F., Xu X., Yin G., Wang S. (2022). m6A Methylation Is Required for Dihydroartemisinin to Alleviate Liver Fibrosis by Inducing Ferroptosis in Hepatic Stellate Cells. Free Radic. Biol. Med..

[179] Zheng Y., Zhao T., Wang J., Jiang R., Huang J., Li W., Wang J. (2022). Curcumol Alleviates Liver Fibrosis through Inducing Autophagy and Ferroptosis in Hepatic Stellate Cells. FASEB J. Off. Publ. Fed. Am. Soc. Exp. Biol..

[180] Yi J., Wu S., Tan S., Qin Y., Wang X., Jiang J., Liu H., Wu B. (2021). Berberine Alleviates Liver Fibrosis through Inducing Ferrous Redox to Activate ROS-Mediated Hepatic Stellate Cells Ferroptosis. Cell Death Discov..

[181] Shi Y., Yan T., Lu X., Li K., Nie Y., Jiao C., Sun H., Li T., Li X., Han D. (2022). Phloridzin Reveals New Treatment Strategies for Liver Fibrosis. Pharmaceuticals.

[182] Choi Y.J., Kim D.H., Kim S.J., Kim J., Jeong S.-I., Chung C.H., Yu K.-Y., Kim S.-Y. (2014). Decursin Attenuates Hepatic Fibrogenesis through Interrupting TGF-Beta-Mediated NAD(P)H Oxidase Activation and Smad Signaling in Vivo and in Vitro. Life Sci..

[183] Que R., Cao M., Dai Y., Zhou Y., Chen Y., Lin L. (2022). Decursin Ameliorates Carbon-Tetrachloride-Induced Liver Fibrosis by Facilitating Ferroptosis of Hepatic Stellate Cells. Biochem. Cell Biol. Biochim. Biol. Cell..

[184] Kil I.S., Bae S.H., Rhee S.G. (2013). Study of the Signaling Function of Sulfiredoxin and Peroxiredoxin III in Isolated Adrenal Gland: Unsuitability of Clonal and Primary Adrenocortical Cells. Methods Enzymol..

[185] Luo P., Liu D., Zhang Q., Yang F., Wong Y.-K., Xia F., Zhang J., Chen J., Tian Y., Yang C. (2022). Celastrol Induces Ferroptosis in Activated HSCs to Ameliorate Hepatic Fibrosis via Targeting Peroxiredoxins and HO-1. Acta Pharm. Sin. B.

[186] Sui M., Jiang X., Chen J., Yang H., Zhu Y. (2018). Magnesium Isoglycyrrhizinate Ameliorates Liver Fibrosis and Hepatic Stellate Cell Activation by Regulating Ferroptosis Signaling Pathway. Biomed. Pharmacother..

[187] Li L., Wang K., Jia R., Xie J., Ma L., Hao Z., Zhang W., Mo J., Ren F. (2022). Ferroportin-Dependent Ferroptosis Induced by Ellagic Acid Retards Liver Fibrosis by Impairing the SNARE Complexes Formation. Redox Biol..

[188] Deng G., Li Y., Ma S., Gao Z., Zeng T., Chen L., Ye H., Yang M., Shi H., Yao X. (2020). Caveolin-1 Dictates Ferroptosis in the Execution of Acute Immune-Mediated Hepatic Damage by Attenuating Nitrogen Stress. Free Radic. Biol. Med..

[189] Li X., Sun R., Liu R. (2019). Natural Products in Licorice for the Therapy of Liver Diseases: Progress and Future Opportunities. Pharmacol. Res..

[190] Huang S., Wang Y., Xie S., Lai Y., Mo C., Zeng T., Kuang S., Zhou C., Zeng Z., Chen Y. (2022). Isoliquiritigenin Alleviates Liver Fibrosis through Caveolin-1-Mediated Hepatic Stellate Cells Ferroptosis in Zebrafish and Mice. Phytomed. Int. J. Phytother. Phytopharm..

[191] Chen S., He Z., Xie W., Chen X., Lin Z., Ma J., Liu Z., Yang S., Wang Y. (2022). Ginsenoside Rh2 Attenuates CDAHFD-Induced Liver Fibrosis in Mice by Improving Intestinal Microbial Composition and Regulating LPS-Mediated Autophagy. Phytomed. Int. J. Phytother. Phytopharm..

[192] Lang Z., Yu S., Hu Y., Tao Q., Zhang J., Wang H., Zheng L., Yu Z., Zheng J. (2023). Ginsenoside Rh2 Promotes Hepatic Stellate Cell Ferroptosis and Inactivation via Regulation of IRF1-Inhibited SLC7A11. Phytomed. Int. J. Phytother. Phytopharm..

[193] Ho C.-H., Huang J.-H., Sun M.-S., Tzeng I.-S., Hsu Y.-C., Kuo C.-Y. (2021). Wild Bitter Melon Extract Regulates LPS-Induced Hepatic Stellate Cell Activation, Inflammation, Endoplasmic Reticulum Stress, and Ferroptosis. Evid.-Based Complement. Altern. Med. ECAM.

[194] Kuo C.-Y., Chiu V., Hsieh P.-C., Huang C.-Y., Huang S.J., Tzeng I.-S., Tsai F.-M., Chen M.-L., Liu C.-T., Chen Y.-R. (2020). Chrysophanol Attenuates Hepatitis B Virus X Protein-Induced Hepatic Stellate Cell Fibrosis by Regulating Endoplasmic Reticulum Stress and Ferroptosis. J. Pharmacol. Sci..

[195] Wang C., Su Z., Xu J.-H., Ko C.-Y. (2023). Danshensu Attenuated Lipopolysaccharide-Induced LX-2 and T6 Cells Activation through Regulation of Ferroptosis. Food Sci. Nutr..

[196] Liu G., Wei C., Yuan S., Zhang Z., Li J., Zhang L., Wang G., Fang L. (2022). Wogonoside Attenuates Liver Fibrosis by Triggering Hepatic Stellate Cell Ferroptosis through SOCS1/P53/SLC7A11 Pathway. Phytother. Res. PTR.

[197] Wu A., Feng B., Yu J., Yan L., Che L., Zhuo Y., Luo Y., Yu B., Wu D., Chen D. (2021). Fibroblast Growth Factor 21 Attenuates Iron Overload-Induced Liver Injury and Fibrosis by Inhibiting Ferroptosis. Redox Biol..

[198] Wei Y., Gao C., Wang H., Zhang Y., Gu J., Zhang X., Gong X., Hao Z. (2023). Mori Fructus Aqueous Extracts Attenuates Liver Injury by Inhibiting Ferroptosis via the Nrf2 Pathway. J. Anim. Sci. Biotechnol..

[199] Wei Y., Wang H., Zhang Y., Gu J., Zhang X., Gong X., Hao Z. (2022). Comprehensive Effect of Carbon Tetrachloride and Reversal of Gandankang Formula in Mice Liver: Involved in Oxidative Stress, Excessive Inflammation, and Intestinal Microflora. Antioxidants.

[200] Tang D., Kroemer G., Kang R. (2023). Ferroptosis in Hepatocellular Carcinoma: From Bench to Bedside. Hepatology.

[201] Bekric D., Ocker M., Mayr C., Stintzing S., Ritter M., Kiesslich T., Neureiter D. (2022). Ferroptosis in Hepatocellular Carcinoma: Mechanisms, Drug Targets and Approaches to Clinical Translation. Cancers.

[202] Casini A., Leone S., Vaccaro R., Vivacqua G., Ceci L., Pannarale L., Franchitto A., Onori P., Gaudio E., Mancinelli R. (2022). The Emerging Role of Ferroptosis in Liver Cancers. Life.

[203] Li X., Meng F., Wang H., Sun L., Chang S., Li G., Chen F. (2024). Iron Accumulation and Lipid Peroxidation: Implication of Ferroptosis in Hepatocellular Carcinoma. Front. Endocrinol..

[204] Nie J., Lin B., Zhou M., Wu L., Zheng T. (2018). Role of Ferroptosis in Hepatocellular Carcinoma. J. Cancer Res. Clin. Oncol..

[205] Zhang L., Li X.-M., Shi X.-H., Ye K., Fu X.-L., Wang X., Guo S.-M., Ma J.-Q., Xu F.-F., Sun H.-M. (2023). Sorafenib Triggers Ferroptosis via Inhibition of HBXIP/SCD Axis in Hepatocellular Carcinoma. Acta Pharmacol. Sin..

[206] Huang W., Chen K., Lu Y., Zhang D., Cheng Y., Li L., Huang W., He G., Liao H., Cai L. (2021). ABCC5 Facilitates the Acquired Resistance of Sorafenib through the Inhibition of SLC7A11-Induced Ferroptosis in Hepatocellular Carcinoma. Neoplasia.

[207] Sun X., Ou Z., Chen R., Niu X., Chen D., Kang R., Tang D. (2016). Activation of the P62-Keap1-NRF2 Pathway Protects against Ferroptosis in Hepatocellular Carcinoma Cells. Hepatology.

[208] Zou C.-G., Banerjee R. (2003). Tumor Necrosis Factor-Alpha-Induced Targeted Proteolysis of Cystathionine Beta-Synthase Modulates Redox Homeostasis. J. Biol. Chem..

[209] Hu Z., Li L., Li M., Zhang X., Zhang Y., Ran J., Li L. (2023). miR-21-5p Inhibits Ferroptosis in Hepatocellular Carcinoma Cells by Regulating the AKT/mTOR Signaling Pathway through MELK. J. Immunol. Res..

[210] Wang Z., Li M., Liu Y., Qiao Z., Bai T., Yang L., Liu B. (2021). Dihydroartemisinin Triggers Ferroptosis in Primary Liver Cancer Cells by Promoting and Unfolded Protein Response-induced Upregulation of CHAC1 Expression. Oncol. Rep..

[211] Su Y., Zhao D., Jin C., Li Z., Sun S., Xia S., Zhang Y., Zhang Z., Zhang F., Xu X. (2021). Dihydroartemisinin Induces Ferroptosis in HCC by Promoting the Formation of PEBP1/15-LO. Oxid. Med. Cell. Longev..

[212] Cui Z., Wang H., Li S., Qin T., Shi H., Ma J., Li L., Yu G., Jiang T., Li C. (2022). Dihydroartemisinin Enhances the Inhibitory Effect of Sorafenib on HepG2 Cells by Inducing Ferroptosis and Inhibiting Energy Metabolism. J. Pharmacol. Sci..

[213] Wong K.H., Yang D., Chen S., He C., Chen M. (2022). Development of Nanoscale Drug Delivery Systems of Dihydroartemisinin for Cancer Therapy: A Review. Asian J. Pharm. Sci..

[214] Su Y., Zhang Z., Lee L.T.O., Peng L., Lu L., He X., Zhang X. (2023). Amphiphilic Dendrimer Doping Enhanced pH-Sensitivity of Liposomal Vesicle for Effective Co-Delivery toward Synergistic Ferroptosis-Apoptosis Therapy of Hepatocellular Carcinoma. Adv. Healthc. Mater..

[215] Huang D., Xu D., Chen W., Wu R., Wen Y., Liu A., Lin L., Lin X., Wang X. (2023). Fe-MnO2 Nanosheets Loading Dihydroartemisinin for Ferroptosis and Immunotherapy. Biomed. Pharmacother..

[216] Liu X., Wu Z., Guo C., Guo H., Su Y., Chen Q., Sun C., Liu Q., Chen D., Mu H. (2022). Hypoxia Responsive Nano-Drug Delivery System Based on Angelica Polysaccharide for Liver Cancer Therapy. Drug Deliv..

[217] Li Z.-J., Dai H.-Q., Huang X.-W., Feng J., Deng J.-H., Wang Z.-X., Yang X.-M., Liu Y.-J., Wu Y., Chen P.-H. (2021). Artesunate Synergizes with Sorafenib to Induce Ferroptosis in Hepatocellular Carcinoma. Acta Pharmacol. Sin..

[218] Yang C., Lu T., Liu M., Yuan X., Li D., Zhang J., Zhou L., Xu M. (2023). Tiliroside Targets TBK1 to Induce Ferroptosis and Sensitize Hepatocellular Carcinoma to Sorafenib. Phytomed. Int. J. Phytother. Phytopharm..

[219] Hu Z., Zhao Y., Li L., Jiang J., Li W., Mang Y., Gao Y., Dong Y., Zhu J., Yang C. (2023). Metformin Promotes Ferroptosis and Sensitivity to Sorafenib in Hepatocellular Carcinoma Cells via ATF4/STAT3. Mol. Biol. Rep..

[220] Li H., Yu Y., Liu Y., Luo Z., Law B.Y.K., Zheng Y., Huang X., Li W. (2022). Ursolic Acid Enhances the Antitumor Effects of Sorafenib Associated with Mcl-1-Related Apoptosis and SLC7A11-Dependent Ferroptosis in Human Cancer. Pharmacol. Res..

[221] Elkateb A.S., Nofal S., Ali S.A., Atya H.B. (2023). Camptothecin Sensitizes Hepatocellular Carcinoma Cells to Sorafenib- Induced Ferroptosis Via Suppression of Nrf2. Inflammation.

[222] Zhang Y., Tan Y., Liu S., Yin H., Duan J., Fan L., Zhao X., Jiang B. (2023). Implications of Withaferin A for the Metastatic Potential and Drug Resistance in Hepatocellular Carcinoma Cells via Nrf2-Mediated EMT and Ferroptosis. Toxicol. Mech. Methods.

[223] Xiu Z., Li Y., Fang J., Han J., Li S., Li Y., Yang X., Song G., Li Y., Jin N. (2023). Inhibitory Effects of Esculetin on Liver Cancer Through Triggering NCOA4 Pathway-Mediation Ferritinophagy in Vivo and in Vitro. J. Hepatocell. Carcinoma.

[224] Xiu Z., Zhu Y., Han J., Li Y., Yang X., Yang G., Song G., Li S., Li Y., Cheng C. (2022). Caryophyllene Oxide Induces Ferritinophagy by Regulating the NCOA4/FTH1/LC3 Pathway in Hepatocellular Carcinoma. Front. Pharmacol..

[225] Li J., Yuan J., Li Y., Wang J., Xie Q., Ma R., Wang J., Ren M., Lu D., Xu Z. (2022). D-Borneol Enhances Cisplatin Sensitivity via Autophagy Dependent EMT Signaling and NCOA4-Mediated Ferritinophagy. Phytomedicine.

[226] Zhu Z.-H., Xu X.-T., Shen C.-J., Yuan J.-T., Lou S.-Y., Ma X.-L., Chen X., Yang B., Zhao H.-J. (2023). A Novel Sesquiterpene Lactone Fraction from *Eupatorium chinense* L. Suppresses Hepatocellular Carcinoma Growth by Triggering Ferritinophagy and Mitochondrial Damage. Phytomed. Int. J. Phytother. Phytopharm..

[227] Huang D., Dong X., Li J., Chen Y., Zhou Y., Chen Q., Sun Y. (2023). Steroidal Saponin SSPH I Induces Ferroptosis in HepG2 Cells via Regulating Iron Metabolism. Med. Oncol..

[228] Yan X., Liu Y., Li C., Mao X., Xu T., Hu Z., Zhang C., Lin N., Lin Y., Zhang Y. (2023). Pien-Tze-Huang Prevents Hepatocellular Carcinoma by Inducing Ferroptosis via Inhibiting SLC7A11-GSH-GPX4 Axis. Cancer Cell Int..

[229] Mei F., Liu Y., Zheng S. (2022). Rhamnazin Inhibits Hepatocellular Carcinoma Cell Aggressiveness in Vitro via Glutathione Peroxidase 4-Dependent Ferroptosis. Tohoku J. Exp. Med..

[230] Huang Q., Li J., Ma M., Lv M., Hu R., Sun J., Zhong X., Sun X., Feng W., Ma W. (2023). High-throughput Screening Identification of a Small-molecule Compound That Induces Ferroptosis and Attenuates the Invasion and Migration of Hepatocellular Carcinoma Cells by Targeting the STAT3/GPX4 Axis. Int. J. Oncol..

[231] Peng Y., Li N., Tang F., Qian C., Jia T., Liu J., Xu Y. (2022). Corosolic Acid Sensitizes Ferroptosis by Upregulating HERPUD1 in Liver Cancer Cells. Cell Death Discov..

[232] Jin M., Shi C., Li T., Wu Y., Hu C., Huang G. (2020). Solasonine Promotes Ferroptosis of Hepatoma Carcinoma Cells via Glutathione Peroxidase 4-Induced Destruction of the Glutathione Redox System. Biomed. Pharmacother..

[233] LoBianco F.V., Krager K.J., Johnson E., Godwin C.O., Allen A.R., Crooks P.A., Compadre C.M., Borrelli M.J., Aykin-Burns N. (2022). Parthenolide Induces Rapid Thiol Oxidation That Leads to Ferroptosis in Hepatocellular Carcinoma Cells. Front. Toxicol..

[234] Liu J.-L., Tong L., Luo Y., Gao Y.-J. (2021). [Cryptotanshinone May Induce Ferroptosis of Human Liver Cancer HepG2 Cells]. Zhongguo Yi Xue Ke Xue Yuan Xue Bao.

[235] Chang W.-T., Bow Y.-D., Fu P.-J., Li C.-Y., Wu C.-Y., Chang Y.-H., Teng Y.-N., Li R.-N., Lu M.-C., Liu Y.-C. (2021). A Marine Terpenoid, Heteronemin, Induces Both the Apoptosis and Ferroptosis of Hepatocellular Carcinoma Cells and Involves the ROS and MAPK Pathways. Oxid. Med. Cell. Longev..

[236] Wang Y.-F., Feng J.-Y., Zhao L.-N., Zhao M., Wei X.-F., Geng Y., Yuan H.-F., Hou C.-Y., Zhang H.-H., Wang G.-W. (2023). Aspirin Triggers Ferroptosis in Hepatocellular Carcinoma Cells through Restricting NF-κB P65-Activated SLC7A11 Transcription. Acta Pharmacol. Sin..

[237] He Y., Fang D., Liang T., Pang H., Nong Y., Tang L., Yang Z., Lu C., Han X., Zhao S. (2021). Atractylodin May Induce Ferroptosis of Human Hepatocellular Carcinoma Cells. Ann. Transl. Med..

[238] Zhang Y., Zhang H., Mu J., Han M., Cao Z., Dong F., Wang T., Pan L., Luo W., Li J. (2022). Eupalinolide B Inhibits Hepatic Carcinoma by Inducing Ferroptosis and ROS-ER-JNK Pathway. Acta Biochim. Biophys. Sin..

[239] Xiahou Z., Han J. (2022). Effects of Dehydroabietic Acid on Nontarget Lipidomics and Proteomics of HepG2. Front. Pharmacol..

[240] Liu Z., Ma H., Lai Z. (2023). The Role of Ferroptosis and Cuproptosis in Curcumin against Hepatocellular Carcinoma. Molecules.

[241] Liu Z., Ma H., Lai Z. (2021). Revealing the Potential Mechanism of Astragalus Membranaceus Improving Prognosis of Hepatocellular Carcinoma by Combining Transcriptomics and Network Pharmacology. BMC Complement. Med. Ther..

[242] Wu X., Wang J., Li B., Gong M., Cao C., Song L., Qin L., Wang Y., Zhang Y., Li Y. (2023). Chlorogenic Acid, Rutin, and Quercetin from Lysimachia Christinae Alleviate Triptolide-Induced Multi-Organ Injury in Vivo by Modulating Immunity and AKT/mTOR Signal Pathway to Inhibit Ferroptosis and Apoptosis. Toxicol. Appl. Pharmacol..

[243] Liang Y., Chen S., Han S., Luo L., Shen F., Huang Z. (2023). Toosendanin Induced Hepatotoxicity via Triggering PERK-eIF2α-ATF4 Mediated Ferroptosis. Toxicol. Lett..

[244] Hu M., Zhong Y., Liu J., Zheng S., Lin L., Lin X., Liang B., Huang Y., Xian H., Li Z. (2022). An Adverse Outcome Pathway-Based Approach to Assess Aurantio-Obtusin-Induced Hepatotoxicity. Toxicology.

[245] Li P., Zhang L., Guo Z., Kang Q., Chen C., Liu X., Ma Q., Zhang J., Hu Y., Wang T. (2022). Epimedium Koreanum Nakai-Induced Liver Injury-A Mechanistic Study Using Untargeted Metabolomics. Front. Pharmacol..

